# Efficacy of Dietary Interventions for Irritable Bowel Syndrome: A Systematic Review and Network Meta-Analysis

**DOI:** 10.3390/jcm13247531

**Published:** 2024-12-11

**Authors:** Hossein Haghbin, Fariha Hasan, Manesh Kumar Gangwani, Nurruddinkhodja Zakirkhodjaev, Wade Lee-Smith, Azizullah Beran, Faisal Kamal, Benjamin Hart, Muhammad Aziz

**Affiliations:** 1 Division of Gastroenterology and Hepatology, Bon Secours Mercy Health, Toledo, OH 43608, USA; 2 Department of Internal Medicine, Cooper University Hospital, Camden, NJ 08103, USA; 3 Department of Gastroenterology, University of Arkansas Medical Sciences, Little Rock, AR 72205, USA; 4 Division of Occupational and Environmental Medicine, The University of Texas Health Science Center at Houston, Houston, TX 77021, USA; 5 University of Toledo Libraries, Toledo, OH 43606, USA; 6 Division of Gastroenterology and Hepatology, Indiana University School of Medicine, Indianapolis, IN 46202, USA; 7 Division of Gastroenterology, Thomas Jefferson University, Philadelphia, PA 19107, USA; 8 Division of Gastroenterology, University of Toledo, Toledo, OH 43606, USA

**Keywords:** gluten-free diet, irritable bowel syndrome, low-FODMAP diet, Mediterranean diet

## Abstract

**Introduction:** Irritable bowel syndrome (IBS) is a common condition that alters the quality of life of patients. A variety of dietary interventions have been introduced to address this debilitating condition. The low-FODMAP diet (LFD), gluten-free diet (GFD), and Mediterranean diet are examples showing efficacy. The aim of this network meta-analysis was to compare these interventions to find the best approach. **Methods:** We performed a systematic review of the available literature through 14 March 2024 in the following databases: Embase, PubMed, MEDLINE OVID, Web of Science, CINAHL Plus, and Cochrane Central. We only included randomized controlled trials (RCTs). We used a random effects model and conducted a direct meta-analysis based on the DerSimonian–Laird approach and a network meta-analysis based on the frequentist approach. Mean differences (MDs) with 95% confidence interval (CI) were calculated. The primary outcomes included IBS quality of life (IBS QOL) and IBS symptom severity scale (IBS-SSS). **Results:** We finalized 23 studies including 1689 IBS patients. In the direct meta-analysis, there was no statistically significant difference in any IBS score between GFD and either LFD or standard diet. Meanwhile, the LFD was statistically superior to the standard diet in the IBS-SSS (MD: −46.29, CI: −63.72–−28.86, *p* < 0.01) and IBS QOL (MD: 4.06, CI: 0.72–7.41, *p* = 0.02). On ranking, the Mediterranean diet showed the greatest improvement in IBS-SSS, IBS-QOL, distension, dissatisfaction, and general life interference, followed by the LFD alone or in combination with the GFD. **Conclusions:** We demonstrated the efficacy of dietary interventions such as the LFD and Mediterranean diet in improving IBS. There is a need for large RCTs with head-to-head comparisons, particularly for the Mediterranean diet.

## 1. Introduction

Irritable bowel syndrome (IBS) is a prevalent functional gastrointestinal disorder diagnosed by a constellation of symptoms, including abdominal pain, bloating, and altered bowel habits [1]. The precise etiology of IBS is unknown, and it affects approximately 10–15% of the global population, impacting their quality of life (QOL) [2]. The diagnosis of IBS usually follows Rome IV criteria [3], and pharmacologic treatment is an option [4]. However, pharmacotherapy with bulking agents, anticholinergics, antispasmodics, and antidiarrheals is usually inadequate [5]. As a result, lifestyle changes and dietary interventions are recognized as the cornerstone in managing symptoms. Throughout the various dietary approaches, the low-FODMAP (fermentable oligosaccharides, disaccharides, monosaccharides, and polyols, LFD) diet and the gluten-free diet (GFD) [6] have emerged as promising strategies for relieving IBS-related discomfort [7].

The FODMAP diet consists of short-chain carbohydrates that are poorly absorbed in the duodenum, resulting in fermentation by gut bacteria and exacerbating gas and secretions, contributing to IBS symptoms. The LFD diet centers on restricting foods that contain these specific carbohydrates, which leads to improvement in symptoms [8,9].

Conversely, the GFD diet focuses on the elimination of food with a high gluten content, including wheat, barley, and rye. This approach might help IBS patients with non-celiac gluten sensitivity or coexisting celiac diseases and has been reported to alleviate gastrointestinal symptoms in some IBS patients. However, the role of gluten in IBS pathogenesis and symptoms is not fully unveiled, and evidence regarding GFD effectiveness remains uncertain [2].

Previous systematic reviews evaluating GFD and LFD reached variable conclusions [10,11,12,13]. Moreover, to date, no updated network meta-analysis that incorporates recent randomized controlled trials to facilitate comparisons between different dietary modalities has been performed. Consequently, we undertook a systematic review and network meta-analysis aimed to systematically compare the efficacy of various dietary interventions in the control of IBS symptoms.

## 2. Methods

### 2.1. Search Strategy

We performed a meta-analysis according to the Preferred Reporting Items for Systematic Reviews and Network Meta-Analyses (PRISMA-NMA) statement [14]. We conducted a systematic search of multiple databases up to 14 March 2024: Embase (Embase.com), Medline (OVID MEDLINE ALL), Cochrane Central Register of Controlled Trials (Cochrane Library, Wiley), CINAHL Plus (EBSCOhost, EBSCO), and Web of Science Core Collection (Web of Science, Clarviate). H.H. and M.A. initially designed the search strategy that was further calibrated by W.L-S, an experienced medical librarian. The keywords “irritable bowel syndrome”, “Low FODMAP diet”, “Mediterranean diet”, “mNICE diet”, among others, were used. The search strategy is included in the Supplementary Materials. EndNote 20 (Clarivate, Philadelphia, PA, USA) was used to record the data. Repeated studies were erased before a manual review.

### 2.2. Selection Criteria/Included Interventions

RCTs adhering to the following PICO criteria were analyzed: (1) population: adult patients with IBS; (2) comparison/interventions/control: any dietary intervention including the low-FODMAP diet, gluten-free diet, and/or standard diet; (3) primary outcome scores: IBS quality of life (IBS QOL) and IBS symptom severity scale (IBS-SSS); secondary outcome scores: abdominal distension, dissatisfaction with bowel habits, general life interference, stool consistency, and stool frequency. We excluded abstracts, review articles, observational studies, and case series. We did not implement any language or publication date restrictions of the studies.

### 2.3. Study Screening/Data Extraction

First, the studies were screened manually by title by the authors (H.H. and M.A.). Second, the full texts of the included studies were carefully reviewed. Third, the authors (H.H. and F.H.) collected the pertinent information. In case of any disagreements, they resolved the matter by discussing also with a third author (M.A.). Baseline and outcome data were collected and saved in Microsoft Excel (Version 16.91, Microsoft, Redmond, WA, USA).

### 2.4. Statistical Analysis

Both direct and network meta-analyses were performed. As long as at least two studies compared two diets, they would be eligible for direct meta-analysis using the DerSimonian–Laird approach. Open Meta Analyst (CEBM, Brown University, Providence, RI, USA) and frequentist approach were used for the direct and network meta-analyses, respectively [15]. In cases where there were continuous variables, the analysis generated mean difference (MD), 95% confidence interval (CI), *p*-value, and forest plots. We used the R package ‘Netmeta’ (Bell Labs, Murray Hill, NJ, USA) for network analysis. With the assumption of heterogeneity, we chose a random effects model. As a sensitivity tool for confounding variables, we used the fixed effects regression model. We opted for intention-to-treat (ITT) rather than for a per protocol outcome data. For heterogeneity, we utilized I^2^ statistic, with significant numbers being above 50% [16]. We performed “Net splitting” to find consistency of the results by comparing direct with indirect evidence for each variable. A *p*-value of less than 0.05 was considered statistically significant. Lastly, different diets were ranked using the P-score, and the greater the score, the better the correspondent IBS outcome [17].

### 2.5. Bias Assessment

An assessment of risk of bias for RCTs was conducted through the Cochrane risk of bias tool [18]. For visual qualitative bias assessment, funnel plots were used. For quantitative asymmetry in the funnel plots, Egger’s and Thompson–Sharp regression analyses were utilized [19,20].

## 3. Results

**Figure 1:**
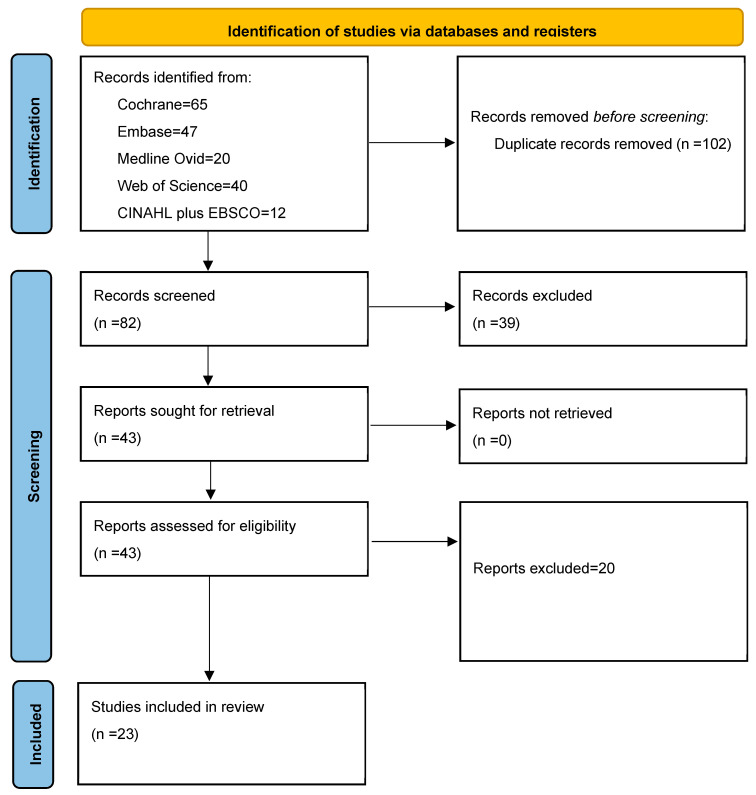
PRISMA flow diagram.

After implementing the inclusion and exclusion criteria, 23 RCTs comprising 1689 patients were included (Figure 1, Table 1) [21,22,23,24,25,26,27,28,29,30,31,32,33,34,35,36,37,38,39,40,41,42,43]. Due to the use of a sequential intervention methodology, Paduano et al.’s study did not require randomization.

Among the articles, 19 compared the LFD to another dietary intervention, one study compared the GFD to the standard diet, two studies compared all three diets, and the remaining ones made comparisons between other diets including the modified National Institute for Health and Clinical Excellence (mNICE) diet, low-lactose diet, low-carb diet, tritordeum-based diet (TBD), Mediterranean diet, LFD in combination with GFD, or LFD with added fiber (Table 1). The articles were published between 2012 and 2024. Information on the studies is reported in Table 1.

### 3.1. Direct Meta-Analysis

#### 3.1.1. Low-FODMAP Diet vs. Standard Diet

Eight studies compared the LFD to the standard diet in terms of IBS-SSS and general life interference score. The LFD showed statistical superiority to the standard diet in regard to IBS-SSS (MD: −46.29, CI: −63.72–−28.86, *p* < 0.01, I^2^ = 55.95%) and general life interference score (MD: −11.46, CI: −13.67–−9.26, *p* < 0.01, I^2^ = 0%, Supplementary Figure S1A,B). Nine studies compared the LFD to the standard diet in terms of dissatisfaction and distension scores. The LFD showed statistical superiority to the standard diet in terms of dissatisfaction score (MD: −10.37, CI: −13.84–−6.9, *p* < 0.01, I^2^ = 32.26%) and distension score (MD: −10.82, CI: −16.35–−5.3, *p* < 0.01, I^2^ = 72.41%, Supplementary Figure S1C,D). Furthermore, the LFD was superior to the standard diet in regard to IBS QOL (four studies, MD: 4.06, CI: 0.72–7.41, *p* = 0.02, I^2^ = 0%) and stool frequency (six studies, MD: −0.33, CI: −0.59–−0.06, *p* = 0.02, I^2^ = 77.3%, Supplementary Figure S1E,F). Stool consistency did not show a statistically significant difference between the LFD and the standard diet (four studies, MD: −0.15, CI: −0.44–0.14, *p* = 0.31, I^2^ = 52.42%) (Supplementary Figure S1G).

#### 3.1.2. Gluten-Free Diet vs. Standard Diet

Three studies made a direct head-to-head comparison between the GFD and the standard diet. In the comparison between the GFD and the standard diet, two studies reported differences for components of the IBS-SSS, with no statistical significance. These included dissatisfaction score (MD: −2.57, CI: −12.99–7.85, *p* = 0.63, I^2^ = 0%), distension score (MD: −5.89, CI: −15.97–4.19, *p* = 0.25, I^2^ = 0%), and general life interference score (MD: −3.71, CI: −13.94–6.53, *p* = 0.48, I^2^ = 0%, Supplementary Figure S2A–C). In terms of IBS QOL, no statistically significant difference was seen between the GFD and the standard diet (MD: 3.03, CI: −8.58–14.63, p = 0.61, I^2^ = 70.22%, Supplementary Figure S2D).

#### 3.1.3. Gluten-Free Diet vs. Low-FODMAP Diet

Three studies made a direct head-to-head comparison between GFD and LFD. Data regarding IBS QOL were the only available for a head-to-head comparison between LFD and GFD, with no statistically significant differences between the two dietary interventions (MD: 1.72, CI: −5.36–8.80, *p* = 0.63, I^2^ = 24.42%, Supplementary Figure S3).

Due to insufficient head-to-head data, we could not perform a direct meta-analysis on other diets.

### 3.2. Network Meta-Analysis

#### 3.2.1. IBS-SSS

**Figure 2:**
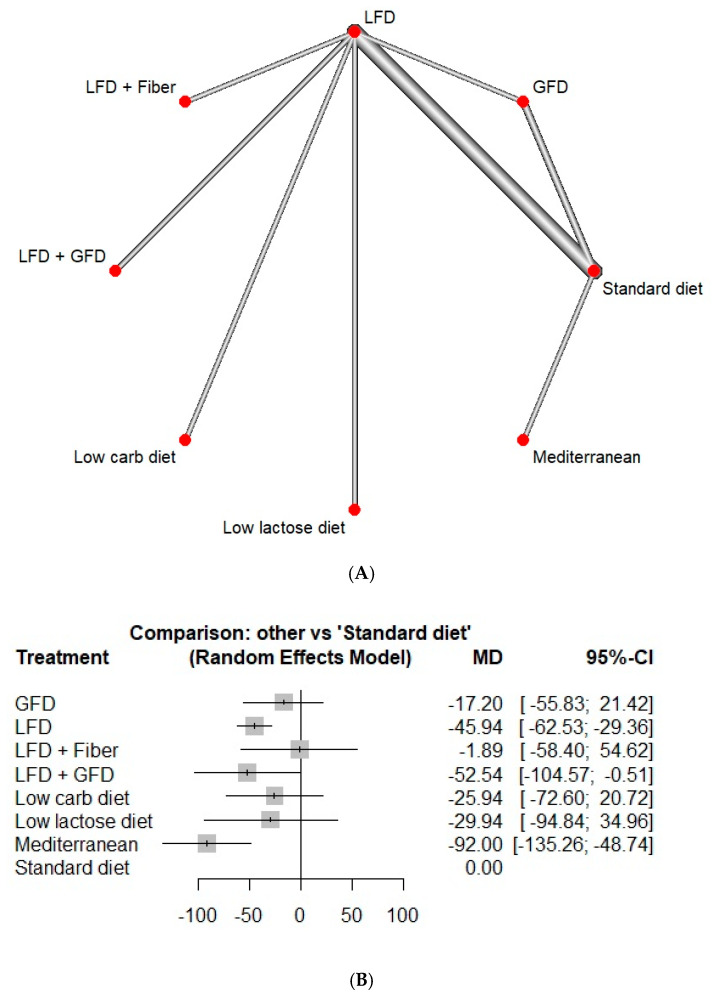
Network meta-analysis of IBS-SSS. (**A**) Network diagram with each line showing a direct comparison of the studies, and the width of the lines indicating the number of studies, (**B**) forest plot with standard diet as a control (GFD: gluten-free diet, IBS-SSS: irritable bowel syndrome symptom severity scale, LFD: low-FODMAP diet).

**Figure 3:**
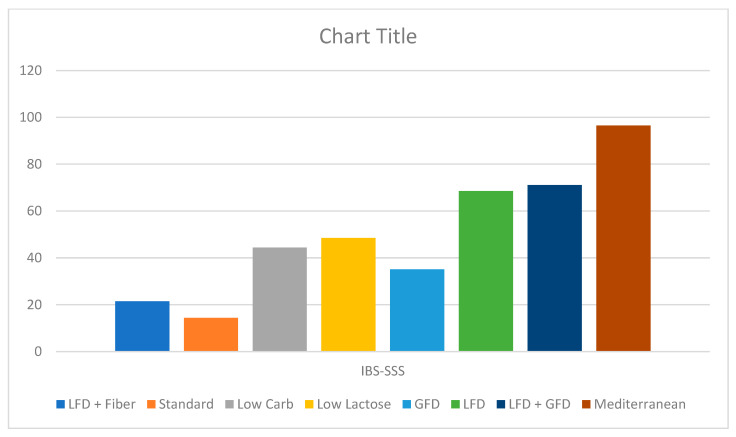
Frequentist approach for ranking with P-score 1–100 for grading, with higher P-scores indicating improvement in IBS-SSS (GFD: gluten-free diet, IBS-SSS: irritable bowel syndrome symptom severity scale, LFD: low-FODMAP diet).

Fourteen studies reported IBS-SSS. Both LFD and Mediterranean diets showed good efficacy in improving IBS-SSS (MD: 45.9, CI:29.4–62.5 and MD: 92.0, CI: 48.7–135.3, respectively, Figure 2). The differences observed when the GFD, low-carb diet, low-lactose diet, and the diet combining LFD with fiber were compared with the standard diet were not statistically significant, with a p-value of less than 0.05 considered as statistically significant. The heterogeneity noted for these outcomes was 49.8% (Figure 2). On Netranking, the Mediterranean diet showed the highest improvement in the IBS-SSS score, followed by the LFD/GFD combination and the LFD (Figure 3). The calculated heterogeneity for this model was 49.8%.

#### 3.2.2. IBS QOL

**Figure 4:**
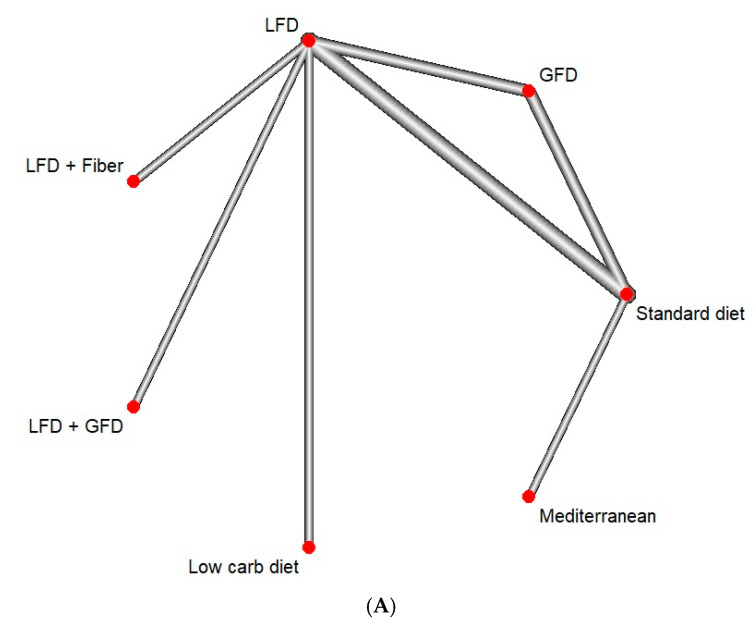

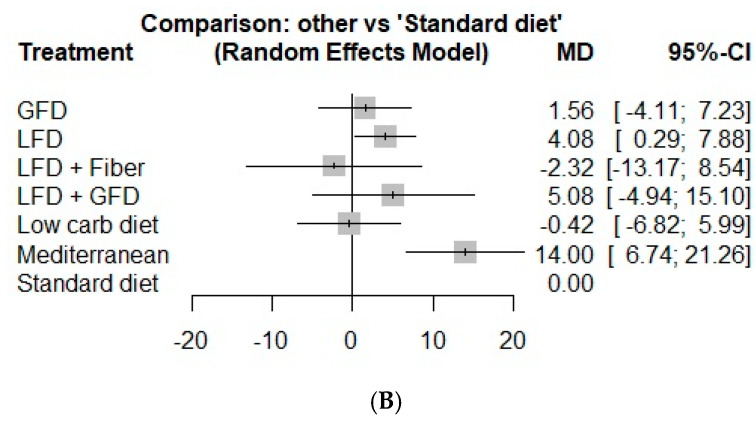
Network meta-analysis of IBS QOL. (**A**) Network diagram, with each line showing a direct comparison of the studies, and the width of the lines indicating the number of studies, (**B**) forest plot, with standard diet as a control (GFD: gluten-free diet, IBS QOL: irritable bowel syndrome quality of life index, LFD: low-FODMAP diet).

**Figure 5:**
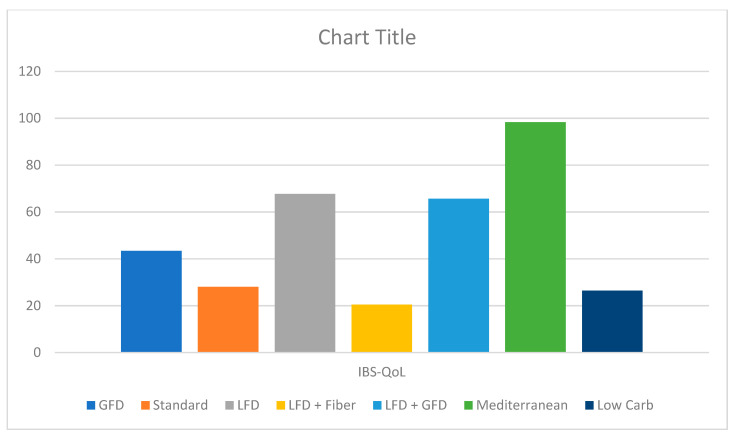
Frequentist approach for ranking with P-score 1–100 for grading, with higher P-scores indicating improvement in IBS QOL (GFD: gluten-free diet, IBS QOL: irritable bowel syndrome quality of life index, LFD: low-FODMAP diet).

Eight studies reported the IBS QOL. Both LFD (MD: 4.1, CI: 0.3–7.9) and Mediterranean diet (MD: 14.0, CI: 6.7–21.3) showed a statistically significant improvement in QOL. The Mediterranean diet was found to be superior to the GFD (MD: 12.4, CI: 3.2–21.7), LFD (MD: 9.9, CI: 1.7–18.1), LFD/fiber combination (MD: 16.3, CI: −3.3–29.4), and low-carb diet (MD: 14.4, CI: 4.7–24.1) (Figure 4). On Netranking, the Mediterranean diet showed the highest improvement in the IBS QOL score, followed by the LFD and LFD/GFD combination (Figure 5). The heterogeneity for this model was 17.3%.

#### 3.2.3. Stool Frequency and Consistency

Eleven studies evaluated the stool frequency, and nine studies reported the stool consistency. The LFD improved the stool frequency compared to the standard diet (MD: 0.3, CI: 0.0–0.6, I2: 75.6%), while the Mediterranean diet improved the stool consistency compared to the standard diet (MD: 0.5, CI: 0.0–1.0, I2: 36.9%) (Supplementary Figures S4 and S5). On ranking, the Mediterranean diet was ranked the highest, followed by the LFD, regarding both stool frequency and consistency (Supplementary Figures S4 and S5).

#### 3.2.4. Abdominal Distension and Dissatisfaction with Bowel Habits

Sixteen studies reported abdominal distension and dissatisfaction with bowel habits. Both LFD and LFD/GFD combination showed improvement in abdominal distension compared to the standard diet (MD: 10.6, CI: 5.3–16.0, I2: 69.1% and MD: 17.2, CI: 0.8–33.6, I2: 69.1%, respectively, Supplementary Figure S6). The LFD/GFD combination showed the highest rank in Netranking (Supplementary Figure S6). In terms of bowel dissatisfaction improvement compared to the standard diet, both LFD (MD: 10.3, CI 7.0–13.6, I2: 26.2%) and Mediterranean diet (MD: 20.0, CI: 7.4–32.6, I2: 26.2%) showed a statistically significant improvement (Supplementary Figure S7). On ranking, the Mediterranean diet ranked first, followed by the LFD (Supplementary Figure S7).

#### 3.2.5. General Life Interference

Fifteen studies reported the effects of different diets on the general life interference score. Compared to the standard diet, the Mediterranean diet (MD: 24.0, CI: 14.2–33.8), low-lactose diet (MD: 13.9, CI: 1.8–26.1), low-carb diet (MD: 8.4, CI: 0.2–16.7), LFD (MD: 11.4, CI: 9.2–13.7), and LFD/GFD combination (MD: 12.0, CI: 2.3–21.8) showed a statistically significant improvement in the general life interference score (I2: 0% for all, Supplementary Figure S8). On ranking, the Mediterranean diet ranked the highest in regard to improvement of general life interference (Supplementary Figure S8). The calculated heterogeneity for this model was a low of 0%.

### 3.3. Bias Assessment

There was a significant risk of bias in blinding of participants and personnel based on the Cochrane risk of bias tool (Supplementary Table S1). This was due to the nature of the interventions, which included dietary interventions and inability to blind per these. Net splitting did not find any statistically significant gaps amongst direct and indirect evidence, which showed consistency in the primary outcomes. (Supplementary Table S2). The publication bias for studies was assessed and is presented in Supplementary Figure S9A–F. The authors found no publication bias after a visual inspection of the funnel plots.

## 4. Discussion

This systematic review, direct meta-analysis, and network meta-analysis provide a comprehensive direct and indirect comparison between various dietary interventions. Our results show the comparative effectiveness of the Mediterranean diet and LFD in alleviating IBS symptoms, particularly abdominal distention, and improving patient quality of life without interfering with their lifestyles.

The FODMAP diet has been observed to increase fermentation and gas production, thus increasing colon volume and leading to abdominal pain, distention, and bloating [33]. For individuals with IBS, who often exhibit visceral hypersensitivity, these symptoms can be exacerbated. The LFD results in a reduction in prebiotic fructans and galactooligosaccharides (GOS) [22,39], thereby decreasing the substrate available for colon fermentation. This reduction is corroborated by a decrease in the release of gases such as hydrogen and methane by the normal flora of the large intestine [44,45,46]. Subsequently, this dietary intervention leads to an improvement in abdominal pain and bloating, ultimately enhancing patients’ quality of life. Furthermore, the LFD has been shown to be a better option compared to other diets in improving stool frequency. This is attributed to the decrease in osmotically active substances, including short-chain fatty acids derived from the fermentation of FODMAPs by bacteria in the alimentary tract [33].

However, our study did not find a statistically significant difference in IBS symptoms, such as abdominal distention, dissatisfaction score, life interference score, with the GFD. The effect of the GFD in IBS patients remains ambiguous, as some studies indicate improvement in symptoms and disease severity assessed by the IBS-SSS score [33]. This improvement may be attributed not solely to the reduction in gluten from the diet but also to the decrease in consumption of packaged foods with fermentable sugars like fructans. Therefore, it might be the combined effect of reducing gluten in addition to other wheat components such as amylase trypsin inhibitors (ATIs), lectin, and fructans that contributes to these outcomes [47,48]. Another aspect to explore is the combination of LFD and GFD. Our analysis indicates improvement in the severity of IBS symptoms with a dietary combination approach. This finding aligns with previous studies such as those of Naseri et al. [49] and Zhang et al. [50], which showed that the combination of LFD and GFD led to a significant improvement in the severity of IBS symptoms. This improvement was attributed to the downregulation of intestinal inflammation and alterations in the gut microbiota.

Many patients with IBS avoid lactose, regardless of whether they have a lactase deficiency. Intolerance to lactose, distinct from lactase deficiency, is common among IBS patients [51,52]. Our study did not find statistically significant differences between the low-lactose diet and the LFD. However, previous research indicated an overall improvement in the severity of IBS symptoms with the LFD compared to the low-lactose diet [27]. This disparity may be attributed to the fact that patients favor less restrictive diets. Some other diets could not be analyzed due to the availability of only inadequate studies. One example is the mNICE diet, which is a modification of the low-FODMAP diet. While the principles of the low-FODMAP diet are applied in this intervention, it also focuses on nutritional adequacy and does not have a restrictive approach. This diet is considered more flexible and advises small frequent meals, portion control, dietary fiber intake, fat reduction, avoidance of alcohol and caffeine, and limited trigger foods [53,54].

Our analysis indicates that the Mediterranean diet was well tolerated. This plant-based dietary approach, characterized by a well-balanced supply of carbohydrates, proteins, lipids, and fibers, encompasses an array of beneficial components including vegetables, fruits, breads, cereals, legumes, nuts, and olive oil [55,56]. This diet plays a crucial role in preventing an excessive FODMAP surplus, thereby mitigating the exacerbation of IBS symptoms. Previous studies have also provided evidence suggesting that this diet not only reduces the risk of cardiovascular diseases and cancer [44,57] but also alleviates the symptoms of depression [58]. Although the Mediterranean diet stands in contrast to the low-FODMAP diet, it improves gastrointestinal symptoms by influencing the microbiome composition [41] as well as gut–brain dysregulation, thus reducing anxiety and depression [41,59].

A notable issue is that substantial dietary restrictions can impact the composition of the gastrointestinal microbiota and its function [44]. Therefore, extreme dietary limitations may lead to nutritional deficiencies in these individuals. Additionally, the implementation of the GFD and LFD is also limited by financial restraints [52]. Many IBS patients have been able to identify dietary triggers by maintaining food diaries, and in most cases find that a specific food that caused symptoms at one time, was well tolerated in others [47]. This supports the argument that IBS patients should adhere to a standard or balanced diet with minimal restrictions [60]. A standard diet, focusing on the equal distribution of FODMAPs in meals, ensures the supply of short-chain fatty acids [61], which serve as the main source of energy for colonic cells and possess anti-carcinogenic and anti-inflammatory characteristics [52] essential for colonic health [62,63].

Limitations of this study include the heterogeneity within the examined studies, with regards to study design and outcome measures, which subsequently might introduce variability in the results. Much of the heterogeneity factors are inherent to studies on nutrition with calculated heterogeneity weight—as reported in the results—being less than high. Furthermore, self-reported outcomes such as subjective symptoms and quality of life are susceptible to recall bias. The lack of blinding of the participants can lead to a greater subjective bias. However, this is like real-life practices of recommending diets to patients. Additionally, there is a lack of data concerning adherence to restrictive diets such as the LFD, leading to challenges in generalizing the results. Moreover, the absence of head-to-head comparisons for diets such as the Mediterranean diet signifies a notable gap in the literature, reminding the need for future research in this area. It is also important to note that many limitations exist with regards to the Mediterranean diet and its definition. While the traditional Mediterranean diet focuses on olive oil as the major source of lipid and on a moderate consumption of fish and poultry, the PREDIMED trial used the MedDiet score, with its key components being the weekly intake of fish, legumes, and nuts and an olive oil consumption of >4 tablespoons per day [56,64]. Hence, a lack of a standardized definition of the Mediterranean diet is a limitation. We recommend a more standardized definition of this diet for future studies.

To conclude, our analysis highlights the low-FODMAP diet’s superiority in the management of IBS symptoms and in improving the quality of life. The Mediterranean diet has also emerged as an alternative dietary approach. Future research should focus on conducting direct comparisons of the Mediterranean diet with the low-FODMAP diet and on exploring the long-term effects of adherence to dietary interventions. Furthermore, adherence to these diets should be explored in controlled settings.

## Figures and Tables

**Table 1:** Baseline information about finalized studies in this meta-analysis.

Study, Year	Design	Group 1	Group 2	Group 3	Group 1, n	Group 2, n	Group 3, n	Duration of Diet	Subtype
Algera [21]	2022	GFD	Standard diet	N/A	20	21	N/A	4 weeks	Both
Bohn [22]	2015	LFD	Standard diet	N/A	38	37	N/A	6 weeks	Both
Eswaran [23]	2016	LFD	mNICE	N/A	50	42	N/A	4 weeks	Both
Goyal [24]	2021	LFD	Standard diet	N/A	51	49	N/A	4 weeks	IBS-D
Guerreiro [25]	2020	LFD	Standard diet	N/A	47	23	N/A	4 weeks	Both
Halmos [26]	2014	LFD	Standard diet	N/A	30	30	N/A	3 weeks	Both
Krieger-Grübel [27]	2020	LFD	Low-lactose diet	N/A	29	29	N/A	4 weeks	Both
Laatikainen [28]	2016	LFD	Standard diet	N/A	80	80	N/A	6 weeks	IBS-D
Liu [29]	2024	LFD	Standard diet	N/A	20	20	N/A	4 weeks	Both
McIntosh [30]	2017	LFD	Standard diet	N/A	18	19	N/A	4 weeks	Both
Mohseni [31]	2022	LFD	LFD + GFD	N/A	26	23	N/A	4 weeks	Both
Nybacka [32]	2024	LFD	Low-carb diet	N/A	96	97	N/A	6 weeks	Both
Paduano [33]	2019	LFD	GFD	Standard diet	42	34	30	4 weeks	IBS-D
Patcharatrakul [34]	2019	LFD	Standard diet	N/A	30	32	N/A	4 weeks	Both
Rej [35]	2022	Standard diet	LFD	GFD	35	33	33	6 weeks	Both
Russo [36]	2022	LFD	TBD	N/A	36	36	N/A	4 weeks	Both
Saadati [37]	2022	LFD + GFD	Standard diet	N/A	15	10	N/A	12 weeks	IBS-D
So [38]	2022	LFD	LFD + Fiber	N/A	26	26	N/A	12 weeks	Both
Staudacher1 [39]	2012	Standard diet	LFD	N/A	22	19	N/A	4 weeks	Both
Staudacher2 [40]	2017	Standard diet	LFD	N/A	53	51	N/A	4 weeks	Both
Staudacher3 [41]	2024	Mediterranean	Standard diet	N/A	29	30	N/A	4 weeks	Both
Wilson [42]	2022	Standard diet	LFD	N/A	23	22	N/A	4 weeks	Both
Zahedi [43]	2018	LFD	Standard diet	N/A	55	55	N/A	6 weeks	Both

Both: IBS-D and IBS-C, GFD: gluten-free diet, LFD: low-FODMAP diet, mNICE: modified National Institute for Health and Clinical Excellence diet, N/A: not applicable, TBD: tritordeum-based diet.

## Supplementary Materials

The following supporting information can be downloaded at: https://www.mdpi.com/article/10.3390/jcm13247531/s1.
