# Supplementary material for: Efficacy of Dietary Interventions for Irritable Bowel Syndrome: A Systematic Review and Network Meta-Analysis
Source: J Clin Med. 2024 Dec 11;13(24):7531. doi: 10.3390/jcm13247531 (PMC11728101; doi:10.3390/jcm13247531)
Supplement: Supplementary file 1 [file jcm-13-07531-s001.zip › jcm-3236345-Supplementary.pdf]

**Supplementary Table S1.** Risk of bias table of randomized controlled trials using Cochrane risk of bias tool.

| Study | Random sequence generation (selection bias) | Allocation concealment (selection bias) | Blinding of participants and personnel (performance bias) | Blinding of outcome assessment (detection bias) | Incomplete outcome data (attrition bias) | Selective reporting (reporting bias) |
|---|---|---|---|---|---|---|
| Algera | Low | Low | Low | Low | Low | Low |
| Bohn | Low | Low | High | High | Low | Low |
| Eswaran | Low | Low | High | High | Low | Low |
| Goyal | Low | Low | High | High | Low | Low |
| Guerreiro | Unclear | Low | High | High | Low | Low |
| Halmos | Low | Low | High | High | Low | Low |
| Krieger-Grübel | Low | Low | High | High | Low | Low |
| Laatikainen | Low | Low | Low | Low | Low | Low |
| Liu | Low | Low | High | High | Low | Low |
| McIntosh | Low | Low | High | High | Low | Low |
| Mohseni | Unclear | Low | Low | Low | Low | Low |
| Nybacka | Low | Low | High | High | Low | Low |
| Paduano | Unclear | Unclear | High | High | Low | Low |
| Patcharatrakul | Unclear | Low | High | High | Low | Low |
| Rej | Low | Low | High | High | Low | Low |

| | | | | | | |
|---|---|---|---|---|---|---|
| Russo | Low | Low | High | High | Low | Low |
| Saadati | Unclear | Unclear | Low | Low | Low | Low |
| So | Low | Low | Unclear | High | High | Low |
| Staudacher1 | Low | Low | High | High | Low | Low |
| Staudacher2 | Low | Low | High | High | Low | Low |
| Staudacher3 | Low | Low | High | High | Low | Low |
| Wilson | Low | Low | Low | Low | Low | Low |
| Zahedi | Low | Low | High | High | Low | Low |

**Supplementary Table S2:** Sensitivity analysis of network meta-analysis of IBS-SSS using netsplit technique.

| Comparison | K | Prop | Nma | Direct | Indir. | Diff | Z | p-value |
|---|---|---|---|---|---|---|---|---|
| GFD:LFD | 1 | 0.57 | 28.74 | 32.00 | 24.45 | 7.55 | 0.18 | 0.8533 |
| GFD:LFD + Fiber | 0 | 0 | -15.31 | . | -15.31 | . | . | . |
| GFD:LFD + GFD | 0 | 0 | 35.34 | . | 35.34 | . | . | . |
| GFD:Low carb diet | 0 | 0 | 8.74 | . | 8.74 | . | . | . |
| GFD:Low lactose diet | 0 | 0 | 12.74 | . | 12.74 | . | . | . |
| GFD:Mediterranean | 0 | 0 | 74.90 | . | 74.80 | . | . | . |
| GFD:Standard diet | 2 | 0.84 | -17.20 | -19.00 | -7.76 | -11.24 | -0.21 | 0.8345 |
| LFD:LFD + Fiber | 1 | 1.00 | -44.05 | -44.05 | . | . | . | . |
| LFD:LFD + GFD | 1 | 1.00 | 6.60 | 6.60 | . | . | . | . |
| LFD:Low carb diet | 1 | 1.00 | -20.00 | -20.00 | . | . | . | . |
| LFD:Low lactose diet | 1 | 1.00 | -16.00 | -16.00 | . | . | . | . |
| LFD:Mediterranean | 0 | 0 | 46.06 | . | 46.06 | . | . | . |
| LFD:Standard diet | 8 | 0.99 | -45.94 | -45.89 | -51.00 | 5.11 | 0.06 | 0.9508 |
| LFD + Fiber:LFD + GFD | 0 | 0 | 50.65 | . | 50.65 | . | . | . |
| LFD + Fiber:Low carb diet | 0 | 0 | 24.05 | . | 24.05 | . | . | . |
| LFD + Fiber:Low lactose diet | 0 | 0 | 28.05 | . | 28.05 | . | . | . |
| LFD + Fiber:Mediterranean | 0 | 0 | 90.11 | . | 90.11 | . | . | . |

| | | | | | | | | |
|---|---|---|---|---|---|---|---|---|
| LFD + Fiber:Standard diet | 0 | 0 | -1.89 | . | -1.89 | . | . | . |
| LFD + GFD:Low carb diet | 0 | 0 | -26.60 | . | -26.60 | . | . | . |
| LFD + GFD:Low lactose diet | 0 | 0 | 22.60 | . | 22.60 | . | . | . |
| LFD + GFD:Mediterranean | 0 | 0 | 39.46 | . | 39.46 | . | . | . |
| LFD + GFD:Standard diet | 0 | 0 | -52.54 | . | -52.54 | . | . | . |
| Low carb diet:Low lactose diet | 0 | 0 | 4.00 | . | 4.00 | . | . | . |
| Low carb diet:Mediterranean | 0 | 0 | 66.06 | . | 66.06 | . | . | . |
| Low carb diet:Standard diet | 0 | 0 | -25.94 | . | -25.94 | . | . | . |
| Low lactose diet:Mediterranean | 0 | 0 | 62.06 | . | 62.06 | . | . | . |
| Low lactose diet:Standard diet | 0 | 0 | -29.94 | . | -29.94 | . | . | . |
| Mediterranean:Standard diet | 1 | 1.00 | -92.00 | -92.00 | . | . | . | . |

k: Number of studies providing direct evidence, prop: Direct evidence proportion, nma: Estimated treatment effect (logOR) in network meta-analysis, direct: Estimated treatment effect (logOR) derived from direct evidence, indir.: Estimated treatment effect (logOR) derived from indirect evidence, Diff: Difference between direct and indirect treatment estimates, z - z-value of test for disagreement (direct versus indirect). (IBS-SSS: irritable bowel syndrome symptoms severity scale)

A.

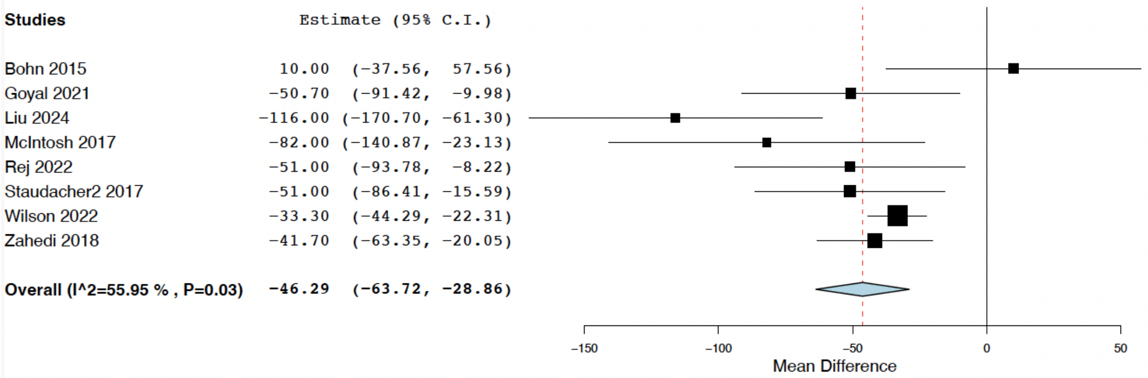

Studies
Estimate (95% C.I.)
Bohn 2015 10.00 (-37.56, 57.56)
Goyal 2021 -50.70 (-91.42, -9.98)
Liu 2024 -116.00 (-170.70, -61.30)
McIntosh 2017 -82.00 (-140.87, -23.13)
Rej 2022 -51.00 (-93.78, -8.22)
Staudacher2 2017 -51.00 (-86.41, -15.59)
Wilson 2022 -33.30 (-44.29, -22.31)
Zahedi 2018 -41.70 (-63.35, -20.05)
Overall (I^2=55.95 % , P=0.03) -46.29 (-63.72, -28.86)
-150
-100
-50
0
50
Mean Difference


B.

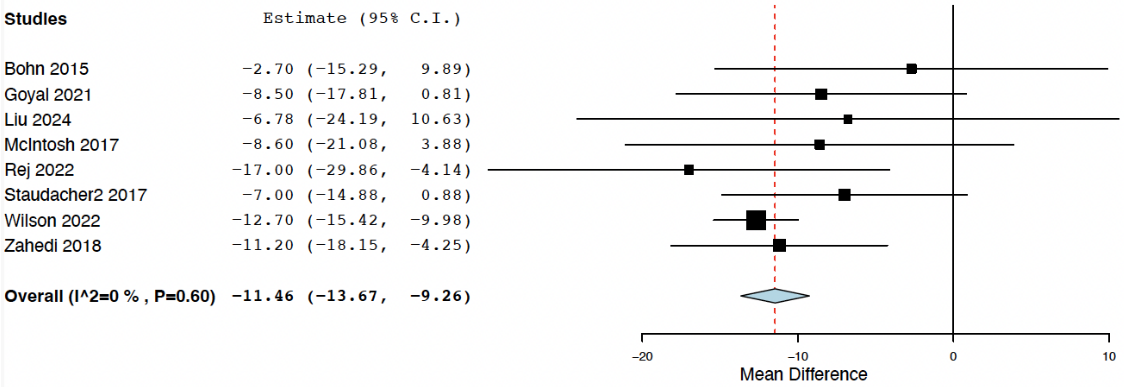

Studies
Estimate (95% C.I.)
Bohn 2015 -2.70 (-15.29, 9.89)
Goyal 2021 -8.50 (-17.81, 0.81)
Liu 2024 -6.78 (-24.19, 10.63)
McIntosh 2017 -8.60 (-21.08, 3.88)
Rej 2022 -17.00 (-29.86, -4.14)
Staudacher2 2017 -7.00 (-14.88, 0.88)
Wilson 2022 -12.70 (-15.42, -9.98)
Zahedi 2018 -11.20 (-18.15, -4.25)
Overall (I^2=0 % , P=0.60) -11.46 (-13.67, -9.26)
-20
-10
0
10
Mean Difference


C.

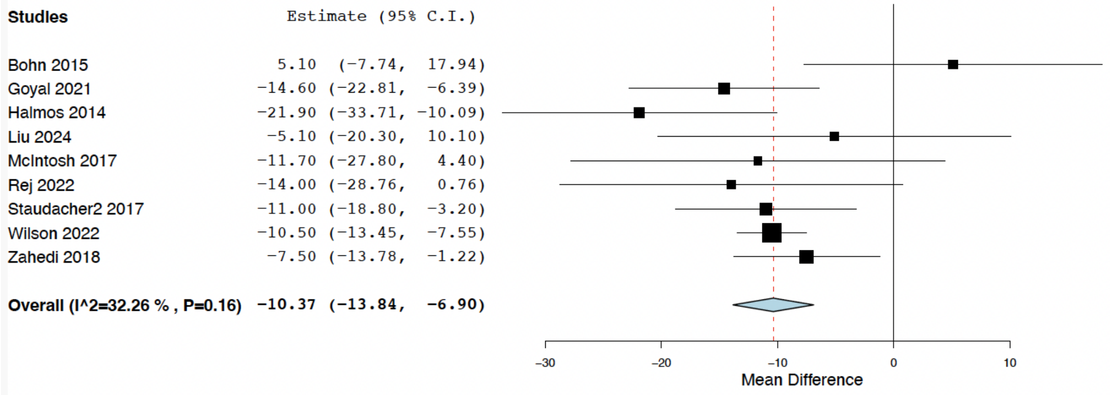

Studies
Estimate (95% C.I.)
Bohn 2015 5.10 (-7.74, 17.94)
Goyal 2021 -14.60 (-22.81, -6.39)
Halmos 2014 -21.90 (-33.71, -10.09)
Liu 2024 -5.10 (-20.30, 10.10)
McIntosh 2017 -11.70 (-27.80, 4.40)
Rej 2022 -14.00 (-28.76, 0.76)
Staudacher2 2017 -11.00 (-18.80, -3.20)
Wilson 2022 -10.50 (-13.45, -7.55)
Zahedi 2018 -7.50 (-13.78, -1.22)
Overall (I^2=32.26 % , P=0.16) -10.37 (-13.84, -6.90)
-30
-20
-10
0
10
Mean Difference

D.

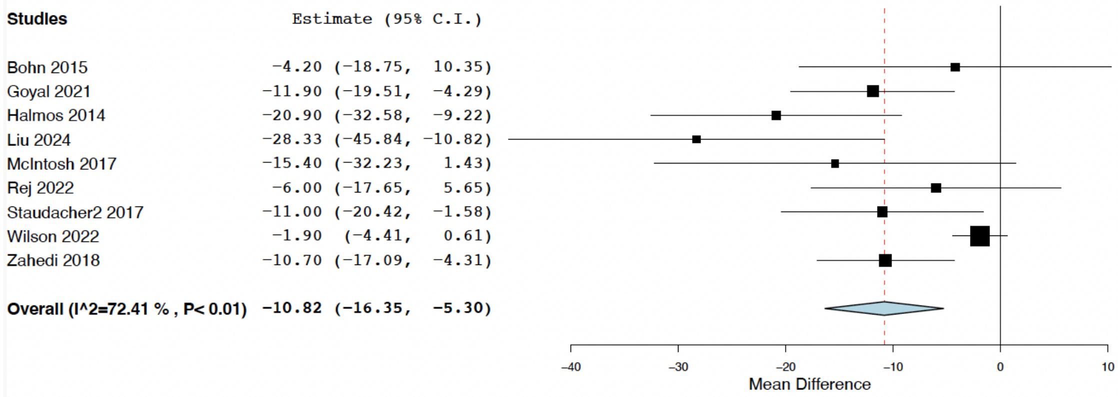
Studies
Estimate (95% C.I.)
Bohn 2015 -4.20 (-18.75, 10.35)
Goyal 2021 -11.90 (-19.51, -4.29)
Halmos 2014 -20.90 (-32.58, -9.22)
Liu 2024 -28.33 (-45.84, -10.82)
McIntosh 2017 -15.40 (-32.23, 1.43)
Rej 2022 -6.00 (-17.65, 5.65)
Staudacher2 2017 -11.00 (-20.42, -1.58)
Wilson 2022 -1.90 (-4.41, 0.61)
Zahedi 2018 -10.70 (-17.09, -4.31)
Overall (I^2=72.41 % , P< 0.01) -10.82 (-16.35, -5.30)
-40
-30
-20
-10
0
10
Mean Difference

E.

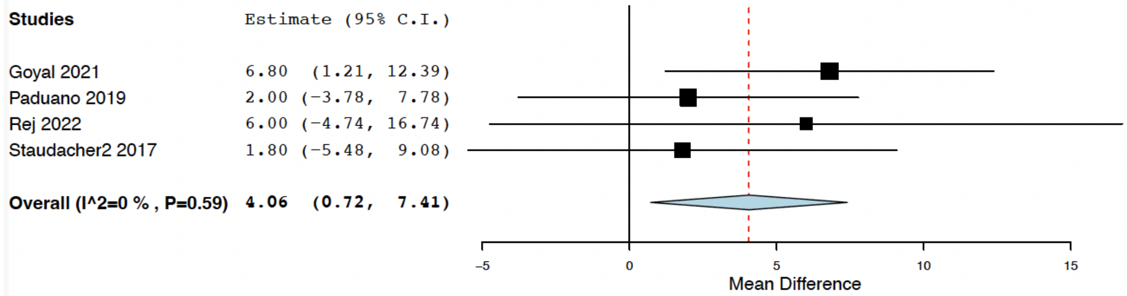
Studies
Estimate (95% C.I.)
Goyal 2021 6.80 (1.21, 12.39)
Paduano 2019 2.00 (-3.78, 7.78)
Rej 2022 6.00 (-4.74, 16.74)
Staudacher2 2017 1.80 (-5.48, 9.08)
Overall (I^2=0 % , P=0.59) 4.06 (0.72, 7.41)
-5
0
5
10
15
Mean Difference

F.

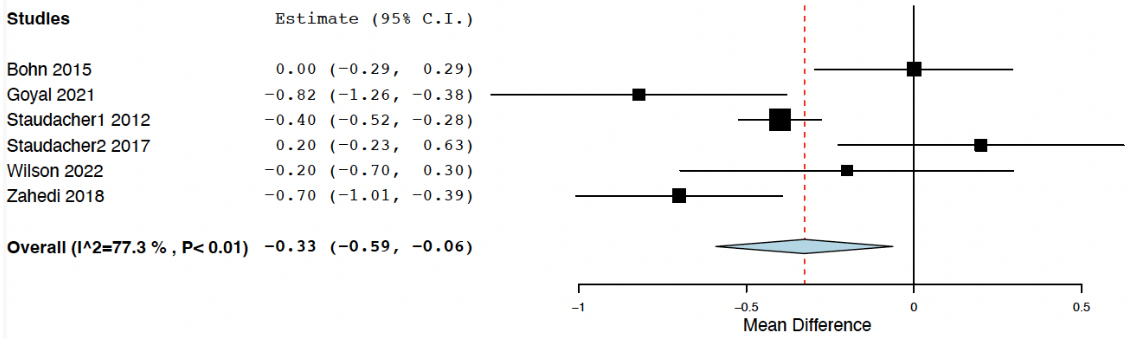
Studies
Estimate (95% C.I.)
Bohn 2015 0.00 (-0.29, 0.29)
Goyal 2021 -0.82 (-1.26, -0.38)
Staudacher1 2012 -0.40 (-0.52, -0.28)
Staudacher2 2017 0.20 (-0.23, 0.63)
Wilson 2022 -0.20 (-0.70, 0.30)
Zahedi 2018 -0.70 (-1.01, -0.39)
Overall (I^2=77.3 % , P< 0.01) -0.33 (-0.59, -0.06)
-1
-0.5
0
0.5
Mean Difference

G.

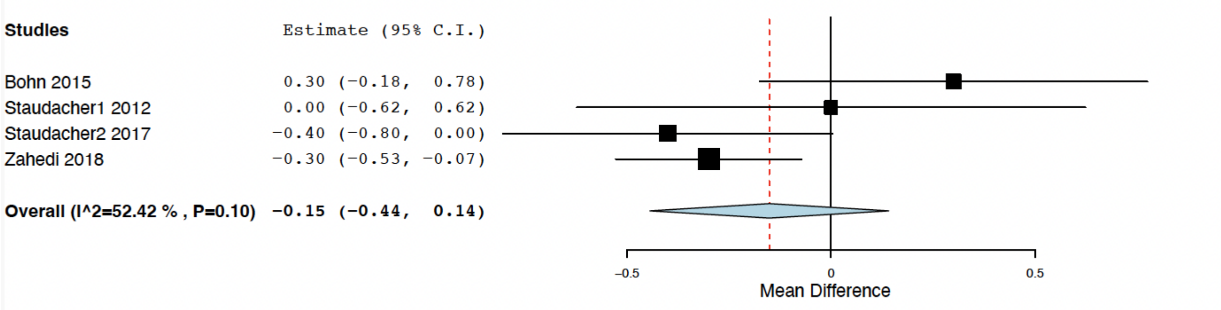


**Supplementary Figure S1.** A. Forest plot comparing IBS-SSS score between low FODMAP diet and standard diet. B. Forest plot comparing general life interference score between low FODMAP diet and standard diet. C. Forest plot comparing dissatisfaction score between low FODMAP diet and standard diet. D. Forest plot comparing distension score between low FODMAP diet and standard diet. E. Forest plot comparing IBS QOL score between low FODMAP diet and standard diet. F. Forest plot comparing stool frequency score between low FODMAP diet and standard diet. G. Forest plot comparing stool consistency score between low FODMAP diet and standard diet. (FODMAP: fermentable oligosaccharides, disaccharides, monosaccharides and polyols, IBS-QOL: irritable bowel syndrome quality of life, IBS-SSS: irritable bowel syndrome symptom severity scale).

A.

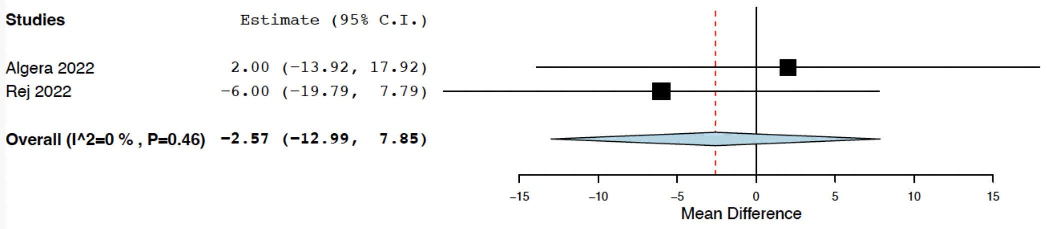


B.

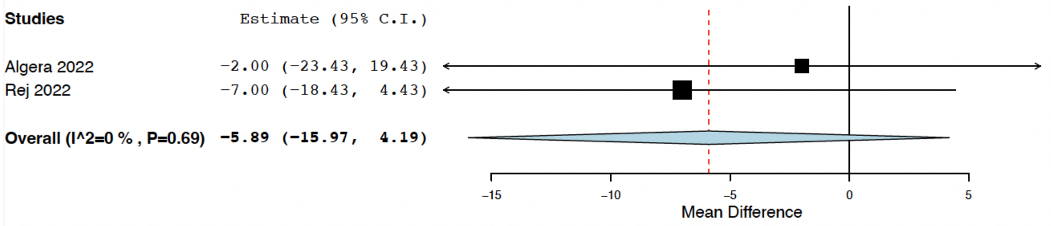


C.

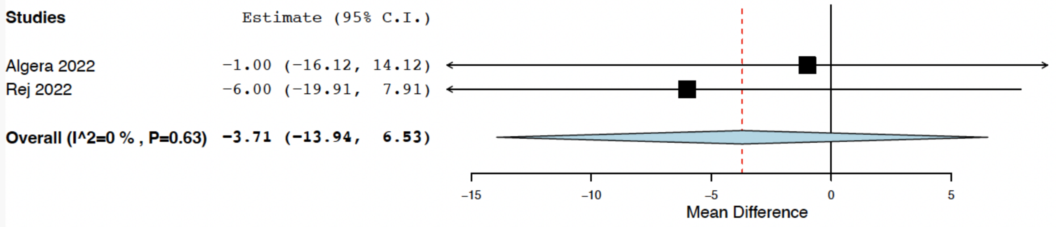


D.

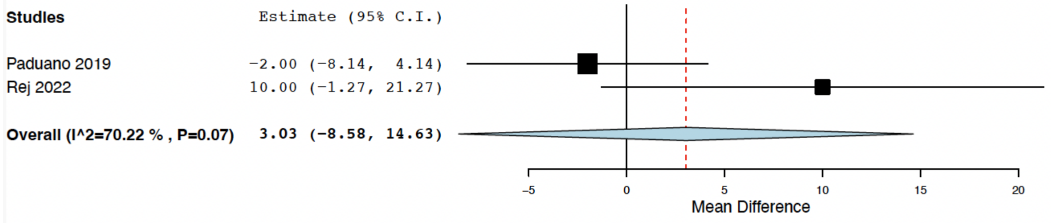


**Supplementary Figure S2.** A. Forest plot comparing dissatisfaction score between gluten-free diet and standard diet. B. Forest plot comparing distension score between gluten-free diet and standard diet. C. Forest plot comparing general life interference score between gluten-free diet and standard diet. D. Forest plot comparing IBS QOL score between gluten-free diet and standard diet. (FODMAP: fermentable oligosaccharides, disaccharides, monosaccharides and polyols, IBS-QOL: irritable bowel syndrome quality of life).

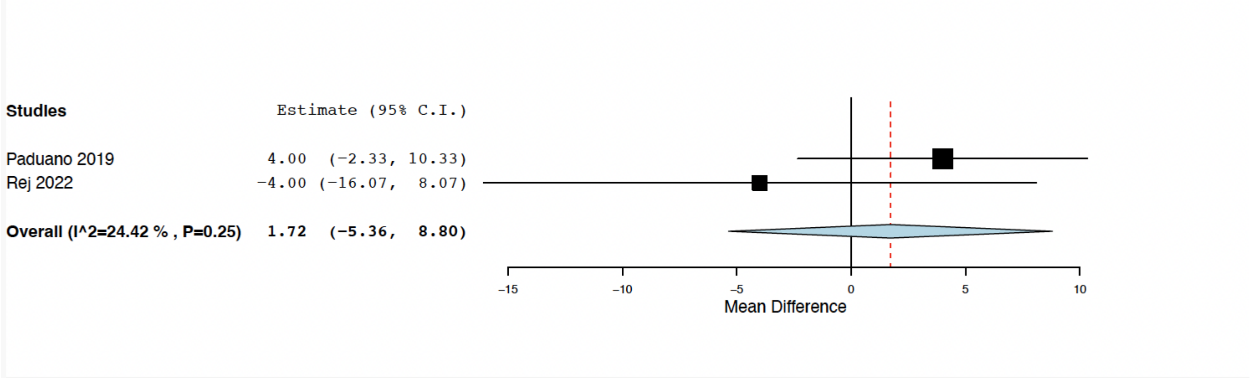


**Supplementary Figure S3.** Forest plot comparing IBS QOL score between low FODMAP diet and gluten-free diet (FODMAP: fermentable oligosaccharides, disaccharides, monosaccharides and polyols, IBS-QOL: irritable bowel syndrome quality of life).

A.

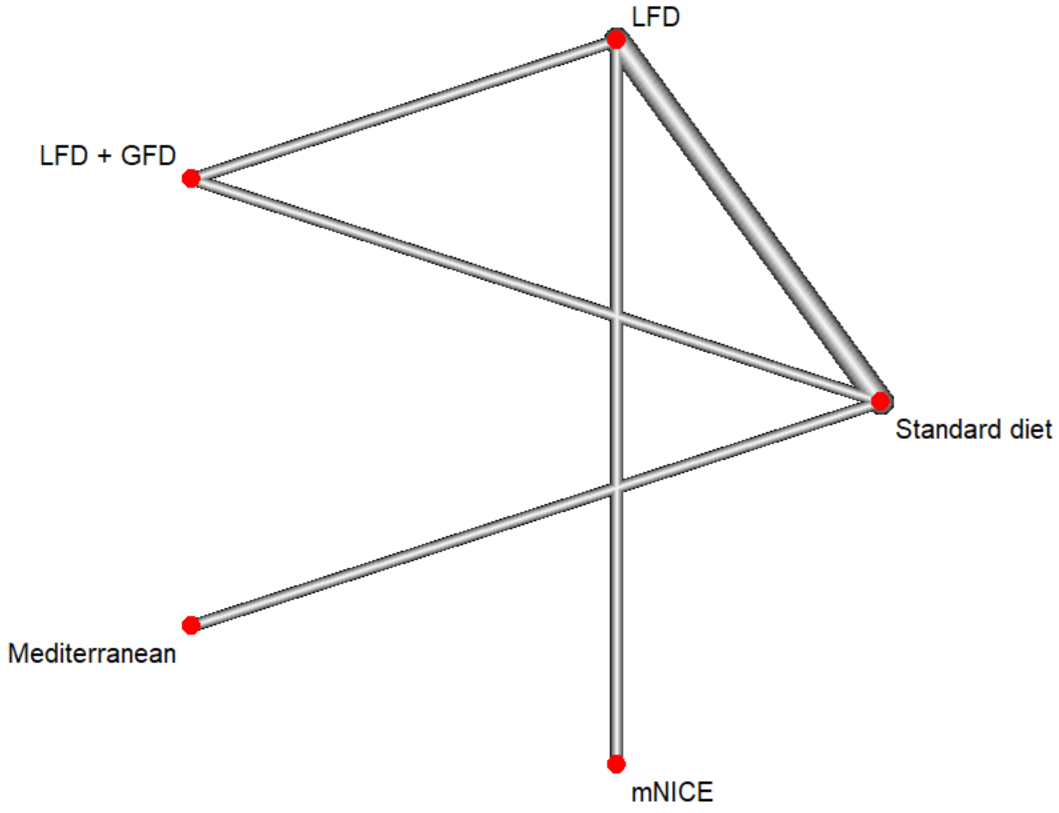
LFD
LFD + GFD
Standard diet
Mediterranean
mNICE

B.

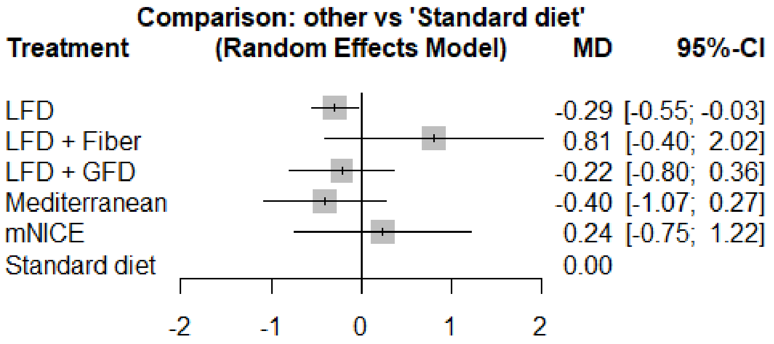
Comparison: other vs 'Standard diet'
Treatment
(Random Effects Model)
MD
95%-CI
LFD
LFD + Fiber
LFD + GFD
Mediterranean
mNICE
Standard diet
-0.29 [-0.55; -0.03]
0.81 [-0.40; 2.02]
-0.22 [-0.80; 0.36]
-0.40 [-1.07; 0.27]
0.24 [-0.75; 1.22]
0.00
-2
-1
0
1
2

C.

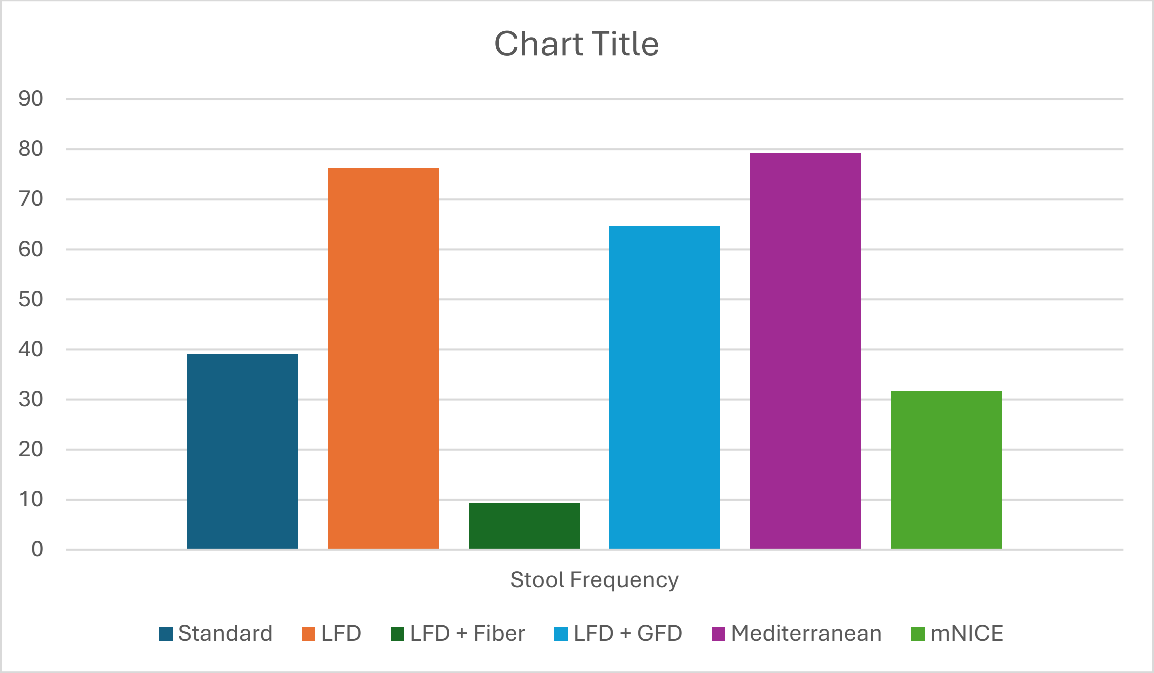


**Supplementary Figure S4:** Stool frequency score using network meta-analysis: (A) Network diagram (the line represents a direct comparison in studies and width of line represents number of studies), (B) Forest plot with standard diet as comparison group C. Ranking using frequentist approach and graded using P-score 1-100. Note: Higher P-score represented improvement in stool frequency (GFD: gluten-free diet, LFD: low FODMAP diet, mNICE: modified National Institute for Health and Clinical Excellence).

A.

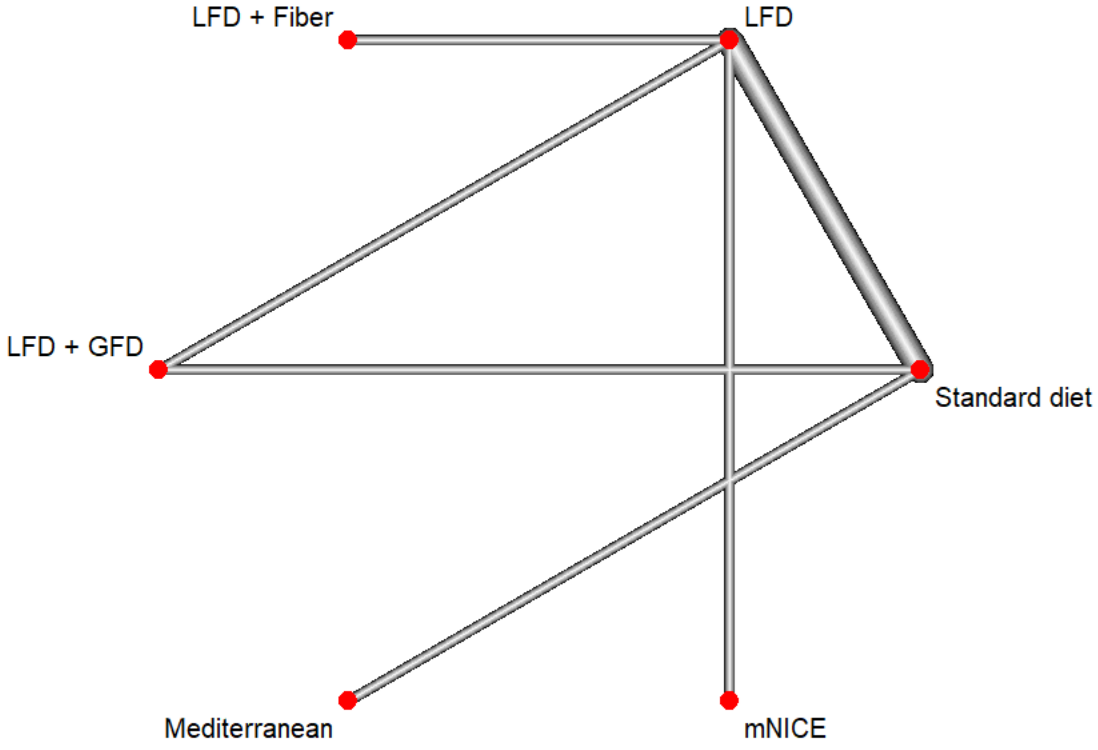
LFD + Fiber
LFD
LFD + GFD
Standard diet
Mediterranean
mNICE

B.

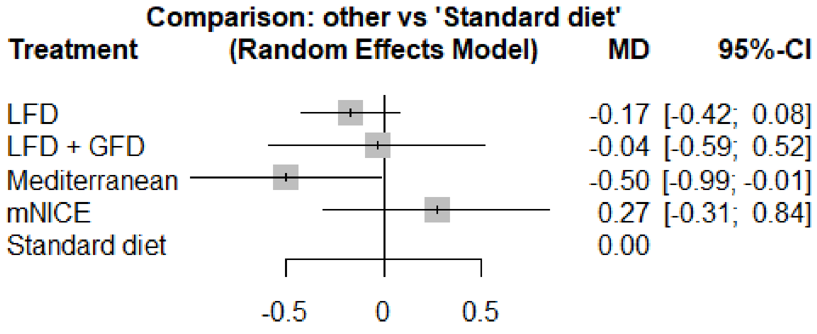
Comparison: other vs 'Standard diet'
Treatment
(Random Effects Model)
MD
95%-CI
LFD
-0.17 [-0.42; 0.08]
LFD + GFD
-0.04 [-0.59; 0.52]
Mediterranean
-0.50 [-0.99; -0.01]
mNICE
0.27 [-0.31; 0.84]
Standard diet
0.00
-0.5
0
0.5

C.

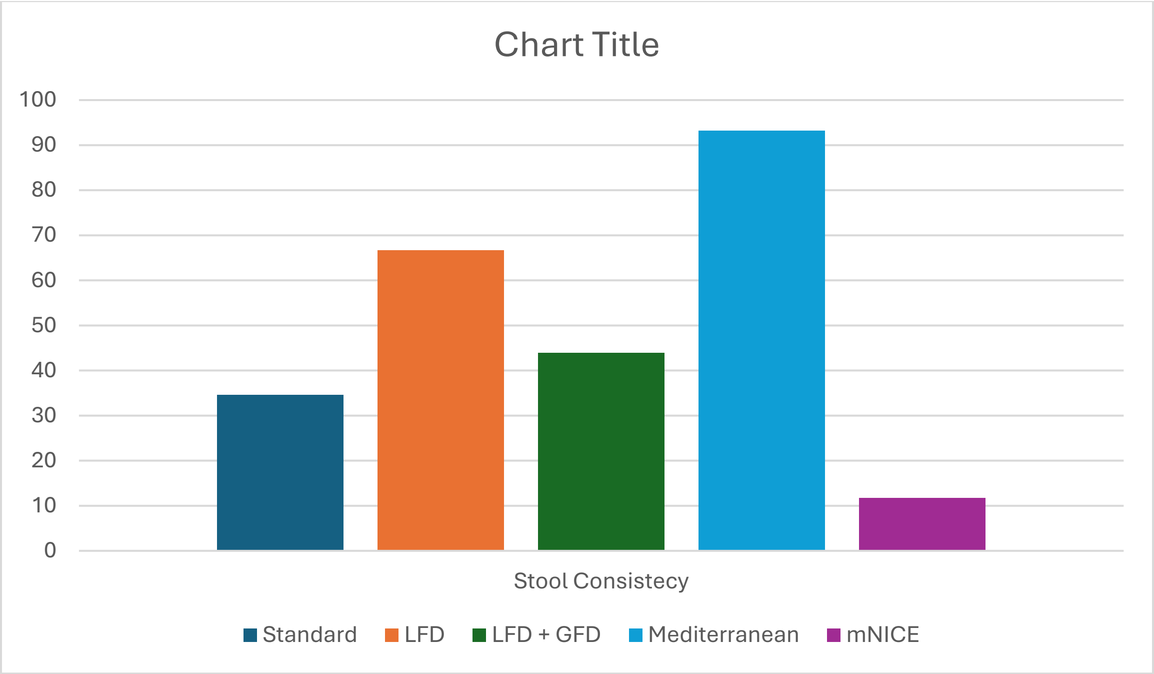


**Supplementary Figure S5:** Stool consistency score using network meta-analysis: (A) Network diagram (the line represents a direct comparison in studies and width of line represents number of studies), (B) Forest plot with standard diet as comparison group C. Ranking using frequentist approach and graded using P-score 1-100. Note: Higher P-score represented improvement in stool consistency (GFD: gluten-free diet, LFD: low FODMAP diet, mNICE: modified National Institute for Health and Clinical Excellence).

A.

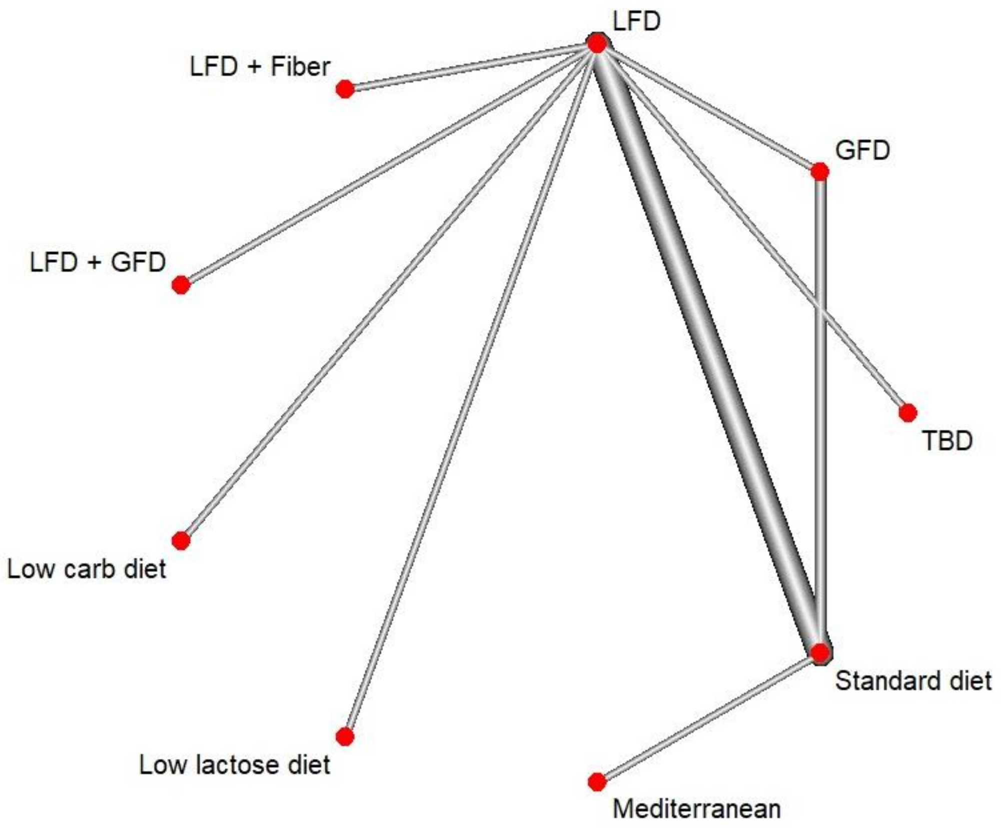
LFD
LFD + Fiber
GFD
LFD + GFD
TBD
Low carb diet
Standard diet
Low lactose diet
Mediterranean

B.

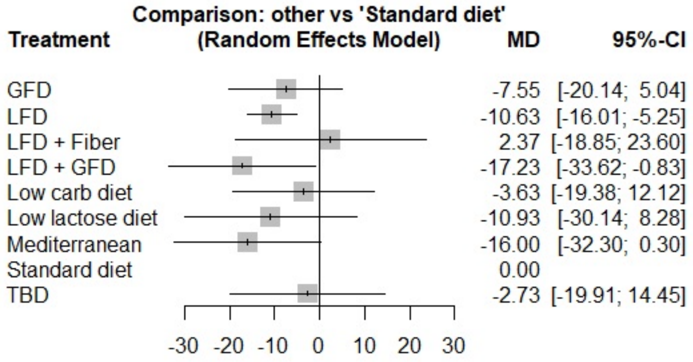
Comparison: other vs 'Standard diet'
(Random Effects Model)
Treatment
MD
95%-CI
GFD -7.55 [-20.14; 5.04]
LFD -10.63 [-16.01; -5.25]
LFD + Fiber 2.37 [-18.85; 23.60]
LFD + GFD -17.23 [-33.62; -0.83]
Low carb diet -3.63 [-19.38; 12.12]
Low lactose diet -10.93 [-30.14; 8.28]
Mediterranean -16.00 [-32.30; 0.30]
Standard diet 0.00
TBD -2.73 [-19.91; 14.45]
-30 -20 -10 0 10 20 30

C.

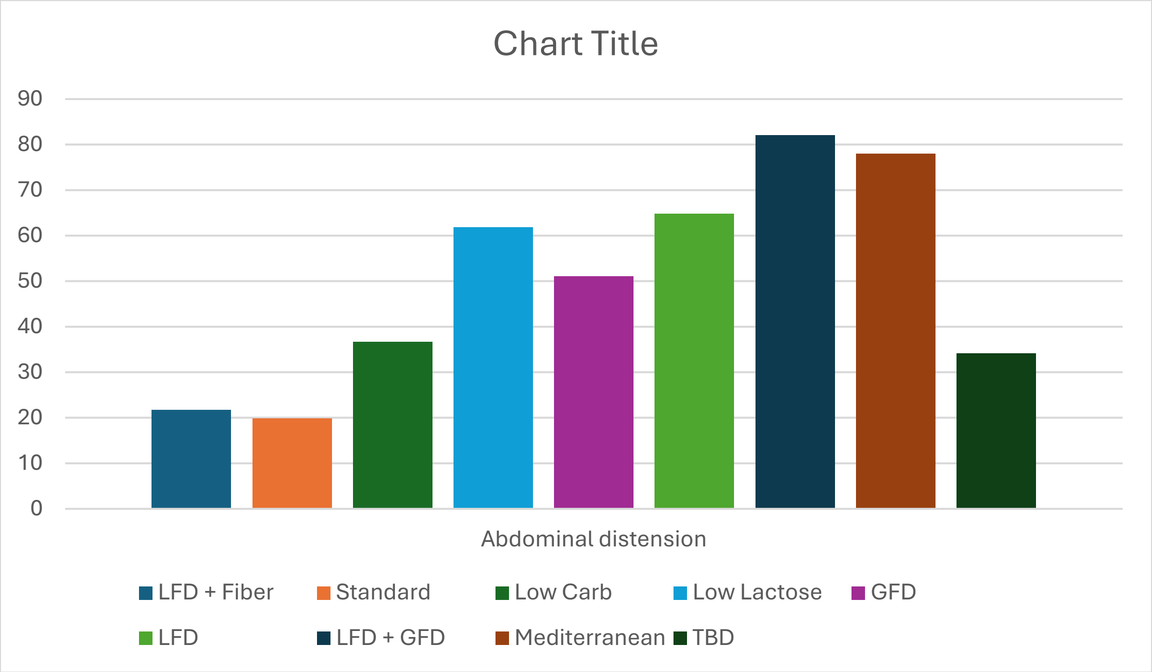


**Supplementary Figure S6:** Abdominal distension score using network meta-analysis: (A) Network diagram (the line represents a direct comparison in studies and width of line represents number of studies), (B) Forest plot with standard diet as comparison group C. Ranking using frequentist approach and graded using P-score 1-100. Note: Higher P-score represented improvement in abdominal distension (GFD: gluten-free diet, LFD: low FODMAP diet, mNICE: modified National Institute for Health and Clinical Excellence).

A.

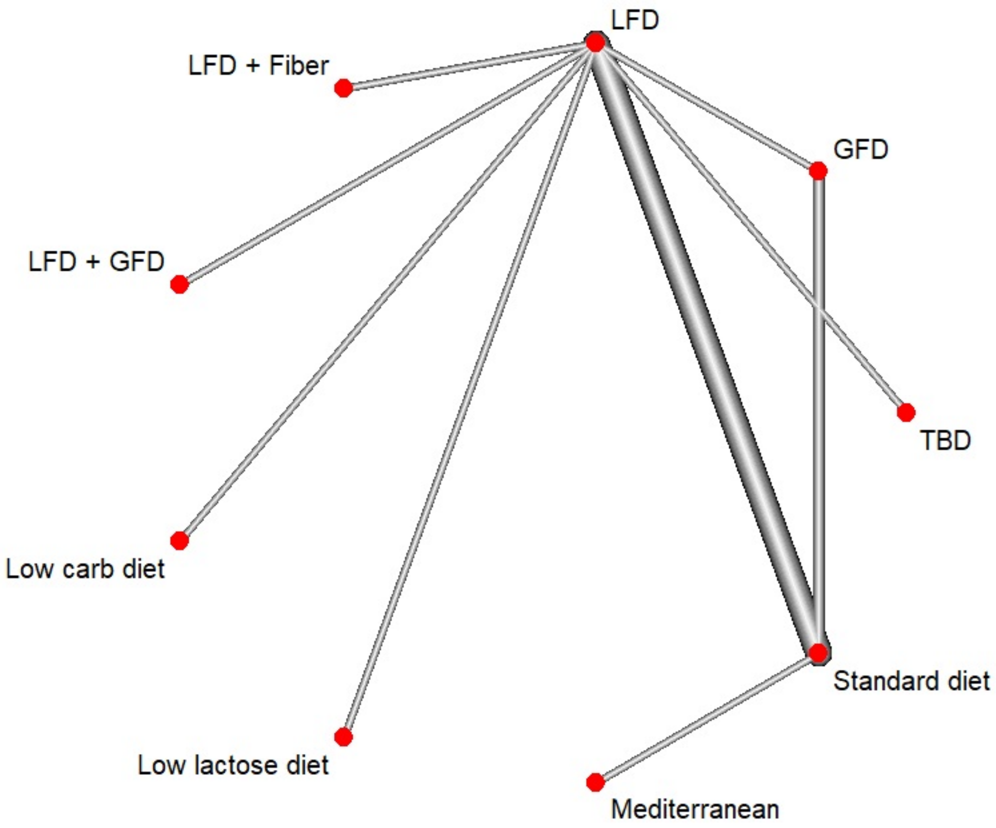

LFD
LFD + Fiber
GFD
LFD + GFD
TBD
Low carb diet
Standard diet
Low lactose diet
Mediterranean


B.

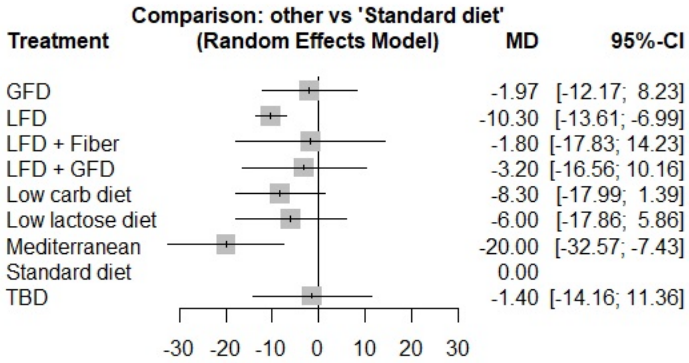

Comparison: other vs 'Standard diet'
(Random Effects Model)
Treatment
MD
95%-CI
GFD -1.97 [-12.17; 8.23]
LFD -10.30 [-13.61; -6.99]
LFD + Fiber -1.80 [-17.83; 14.23]
LFD + GFD -3.20 [-16.56; 10.16]
Low carb diet -8.30 [-17.99; 1.39]
Low lactose diet -6.00 [-17.86; 5.86]
Mediterranean -20.00 [-32.57; -7.43]
Standard diet 0.00
TBD -1.40 [-14.16; 11.36]
-30 -20 -10 0 10 20 30

C.

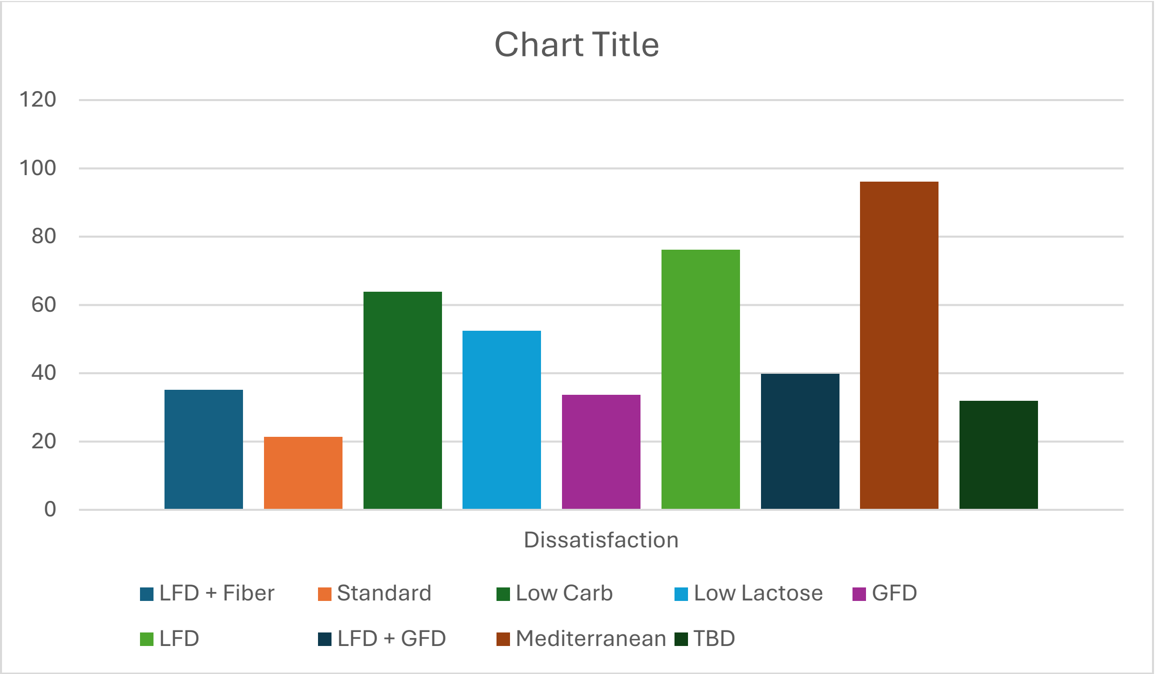


**Supplementary Figure S7:** Bowel dissatisfaction score using network meta-analysis: (A) Network diagram (the line represents a direct comparison in studies and width of line represents number of studies), (B) Forest plot with standard diet as comparison group C. Ranking using frequentist approach and graded using P-score 1-100. Note: Higher P-score represented improvement in bowel dissatisfaction (GFD: gluten-free diet, LFD: low FODMAP diet).

A.

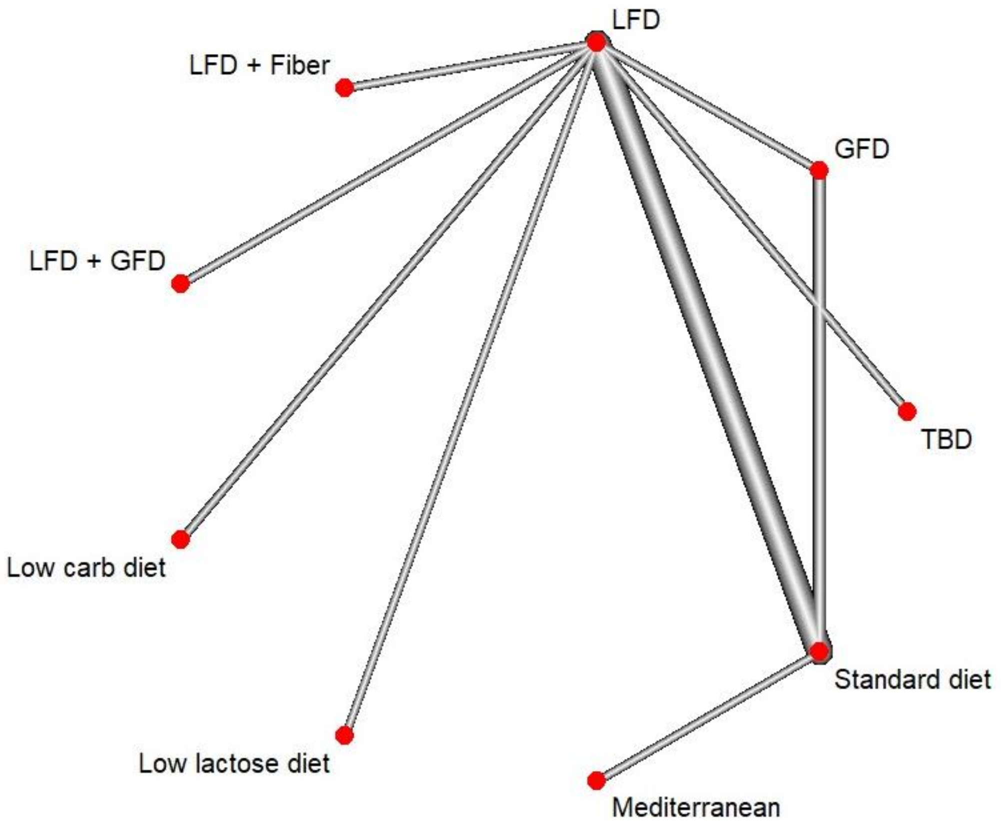
LFD
LFD + Fiber
GFD
LFD + GFD
TBD
Low carb diet
Standard diet
Low lactose diet
Mediterranean

B.

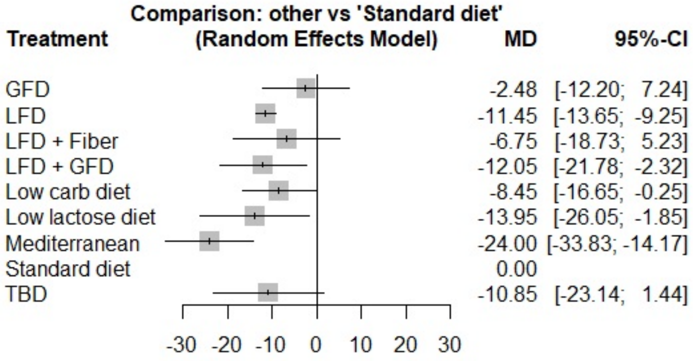
Comparison: other vs 'Standard diet'
(Random Effects Model)
Treatment
MD
95%-CI
GFD -2.48 [-12.20; 7.24]
LFD -11.45 [-13.65; -9.25]
LFD + Fiber -6.75 [-18.73; 5.23]
LFD + GFD -12.05 [-21.78; -2.32]
Low carb diet -8.45 [-16.65; -0.25]
Low lactose diet -13.95 [-26.05; -1.85]
Mediterranean -24.00 [-33.83; -14.17]
Standard diet 0.00
TBD -10.85 [-23.14; 1.44]
-30 -20 -10 0 10 20 30

C.

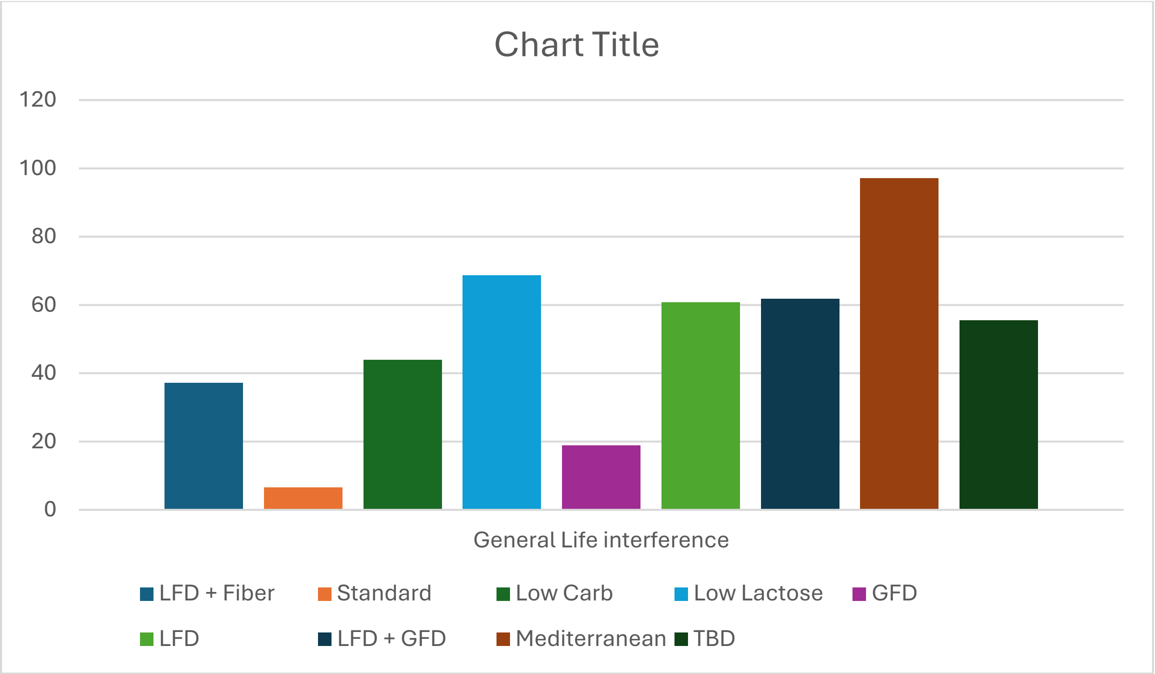


**Supplementary Figure S8:** General life interference score using network meta-analysis: (A) Network diagram (the line represents a direct comparison in studies and width of line represents number of studies), (B) Forest plot with standard diet as comparison group C. Ranking using frequentist approach and graded using P-score 1-100. Note: Higher P-score represented improvement in General life interference (GFD: gluten-free diet, LFD: low FODMAP diet, TBD: Tritordeum-based diet).

A.

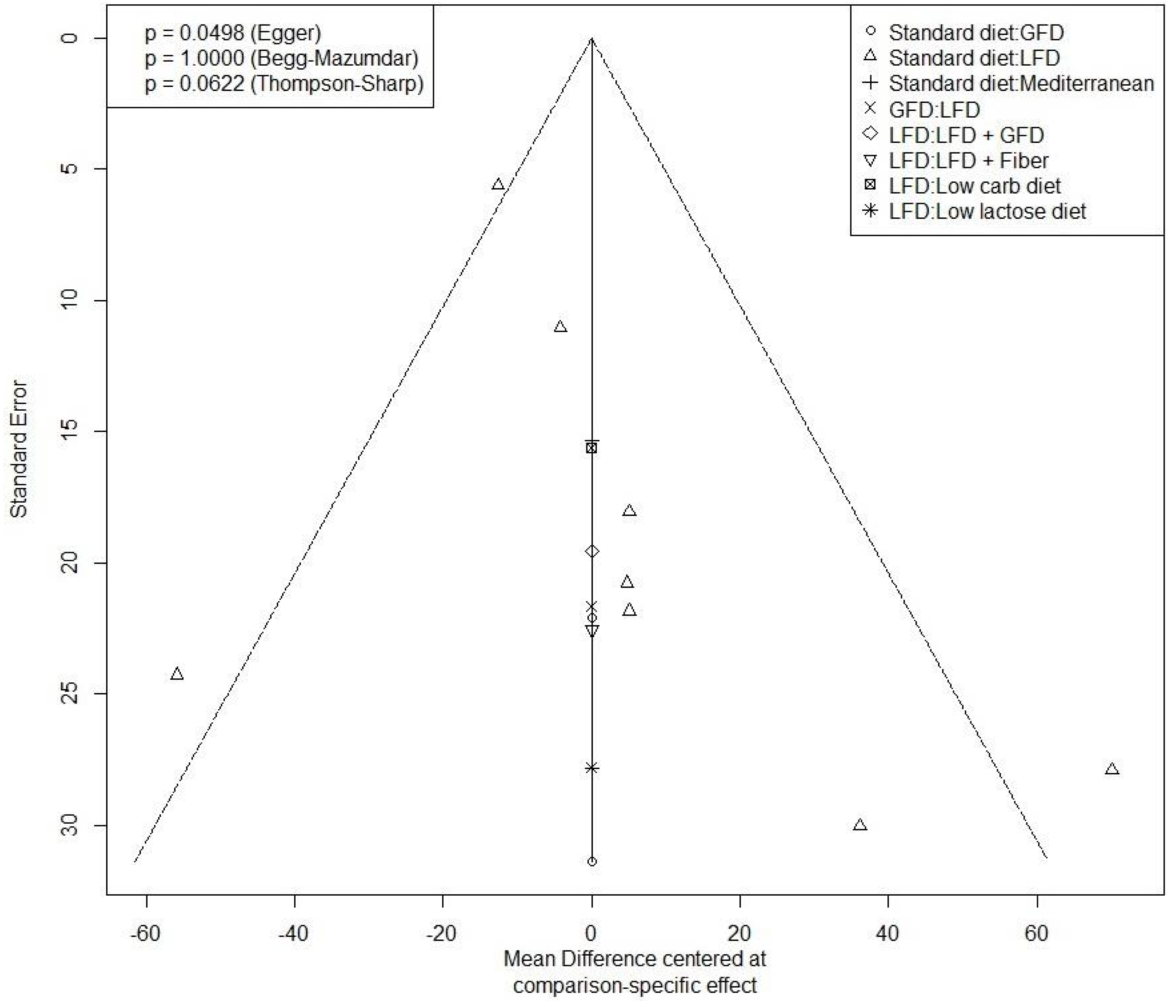
p = 0.0498 (Egger)
p = 1.0000 (Begg-Mazumdar)
p = 0.0622 (Thompson-Sharp)
Standard diet:GFD
Standard diet:LFD
Standard diet:Mediterranean
GFD:LFD
LFD:LFD + GFD
LFD:LFD + Fiber
LFD:Low carb diet
LFD:Low lactose diet
Standard Error
0
5
10
15
20
25
30
-60
-40
-20
0
20
40
60
Mean Difference centered at
comparison-specific effect

B.

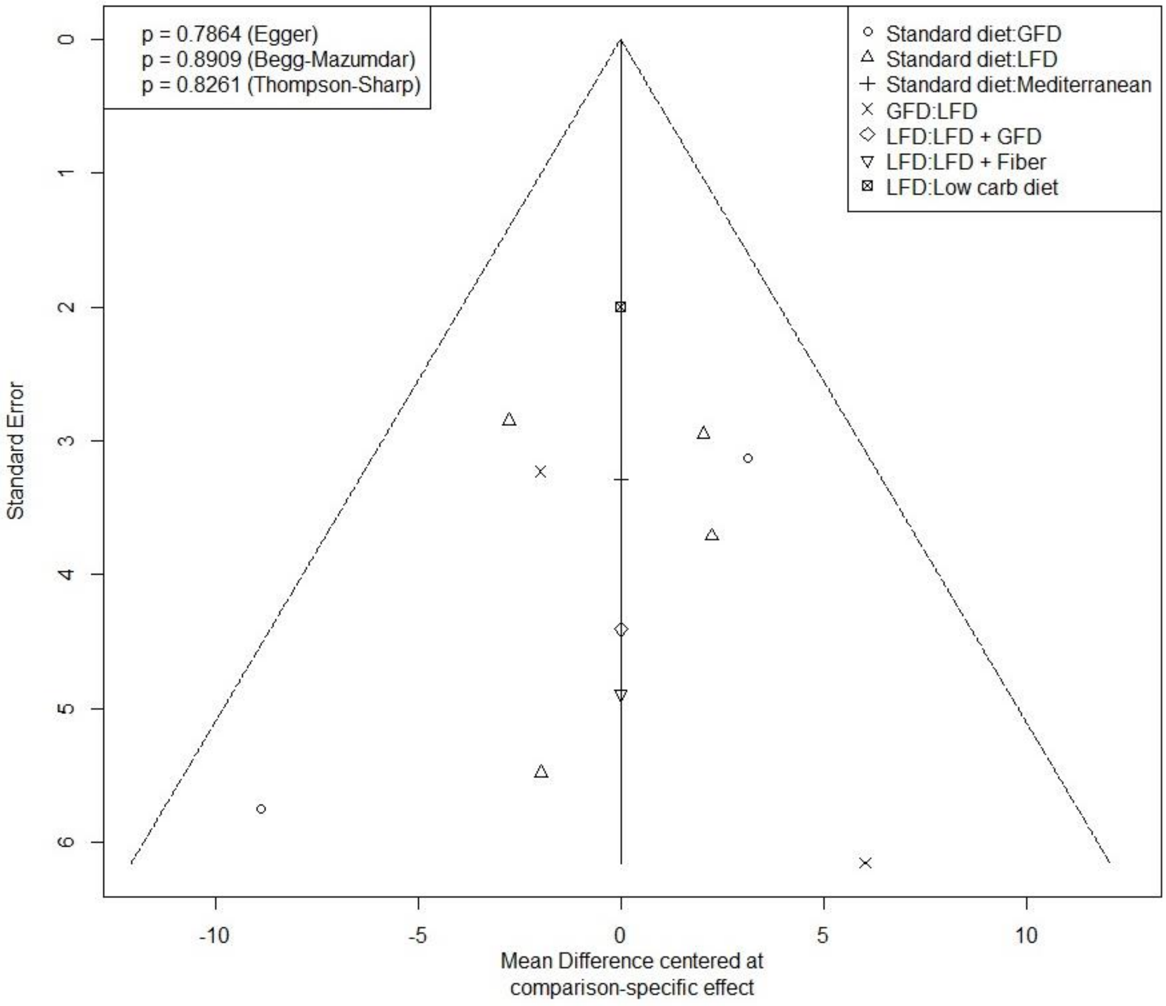

p = 0.7864 (Egger)
p = 0.8909 (Begg-Mazumdar)
p = 0.8261 (Thompson-Sharp)
Standard diet:GFD
Standard diet:LFD
Standard diet:Mediterranean
GFD:LFD
LFD:LFD + GFD
LFD:LFD + Fiber
LFD:Low carb diet
Standard Error
0
1
2
3
4
5
6
-10
-5
0
5
10
Mean Difference centered at comparison-specific effect

C.

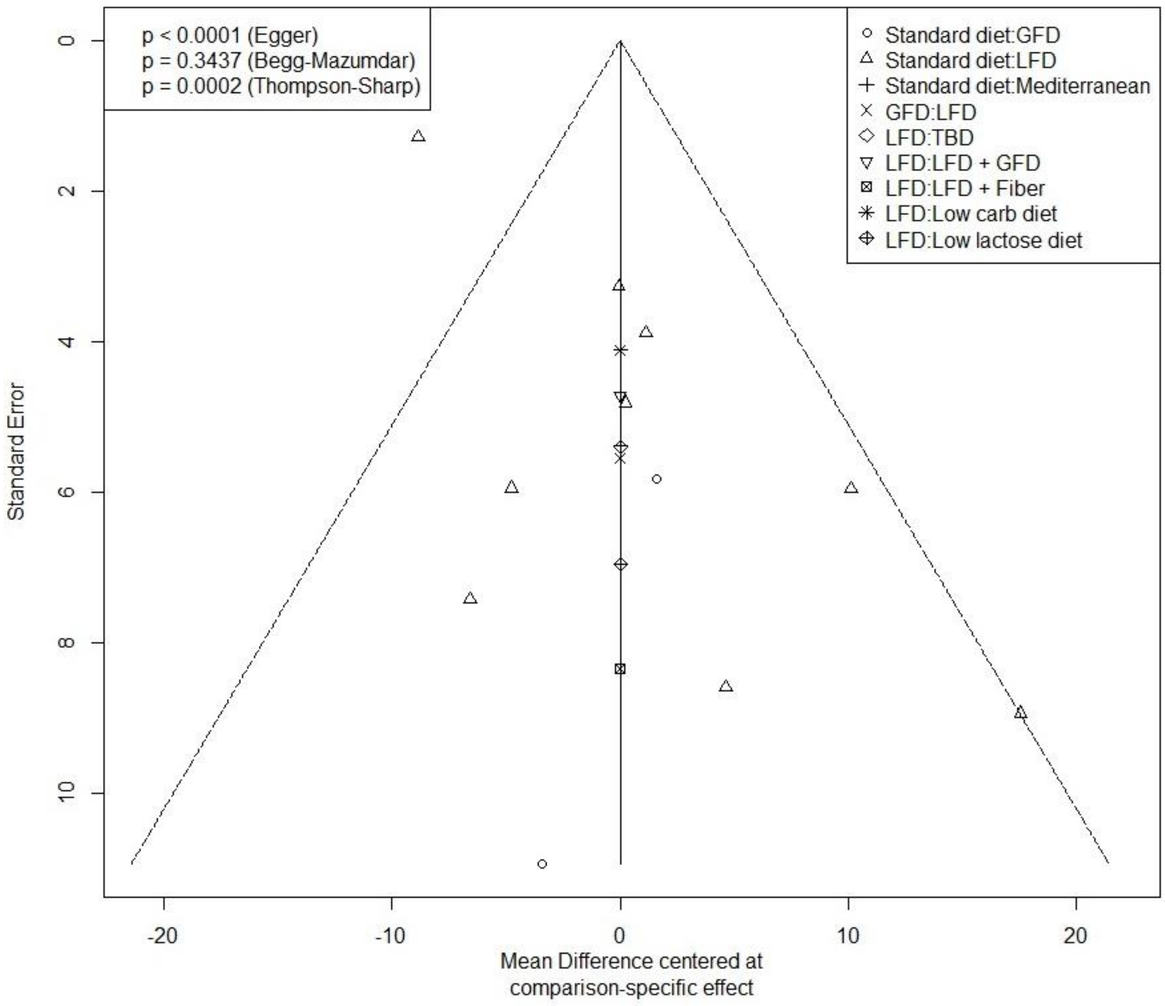
p < 0.0001 (Egger)
p = 0.3437 (Begg-Mazumdar)
p = 0.0002 (Thompson-Sharp)
Standard diet:GFD
Standard diet:LFD
Standard diet:Mediterranean
GFD:LFD
LFD:TBD
LFD:LFD + GFD
LFD:LFD + Fiber
LFD:Low carb diet
LFD:Low lactose diet
Standard Error
0
2
4
6
8
10
-20
-10
0
10
20
Mean Difference centered at comparison-specific effect

D.

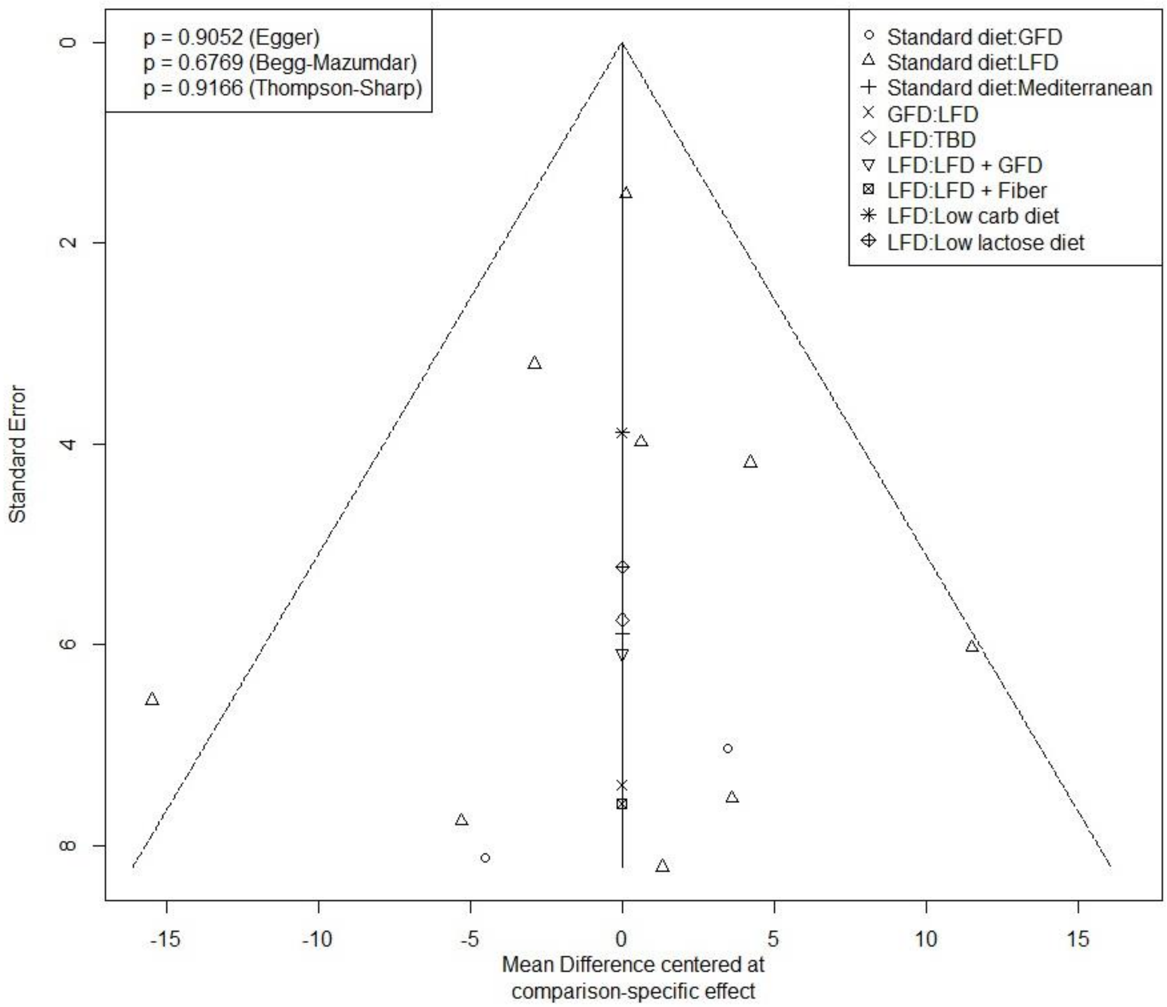
p = 0.9052 (Egger)
p = 0.6769 (Begg-Mazumdar)
p = 0.9166 (Thompson-Sharp)
Standard diet:GFD
Standard diet:LFD
Standard diet:Mediterranean
GFD:LFD
LFD:TBD
LFD:LFD + GFD
LFD:LFD + Fiber
LFD:Low carb diet
LFD:Low lactose diet
Standard Error
0
2
4
6
8
-15
-10
-5
0
5
10
15
Mean Difference centered at
comparison-specific effect

E.

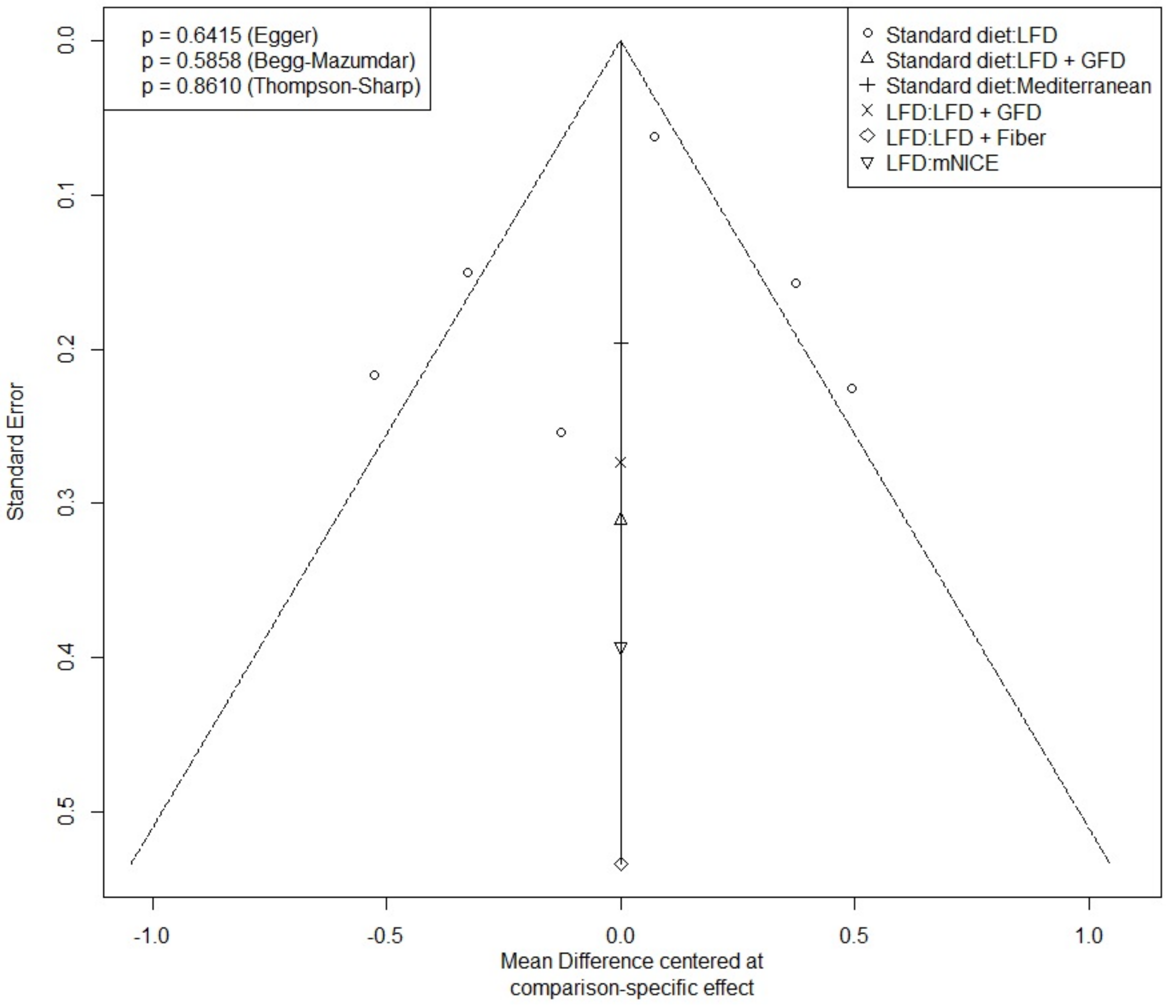
p = 0.6415 (Egger)
p = 0.5858 (Begg-Mazumdar)
p = 0.8610 (Thompson-Sharp)
Standard diet:LFD
Standard diet:LFD + GFD
Standard diet:Mediterranean
LFD:LFD + GFD
LFD:LFD + Fiber
LFD:mNICE
Standard Error
0.0
0.1
0.2
0.3
0.4
0.5
-1.0
-0.5
0.0
0.5
1.0
Mean Difference centered at comparison-specific effect

F.

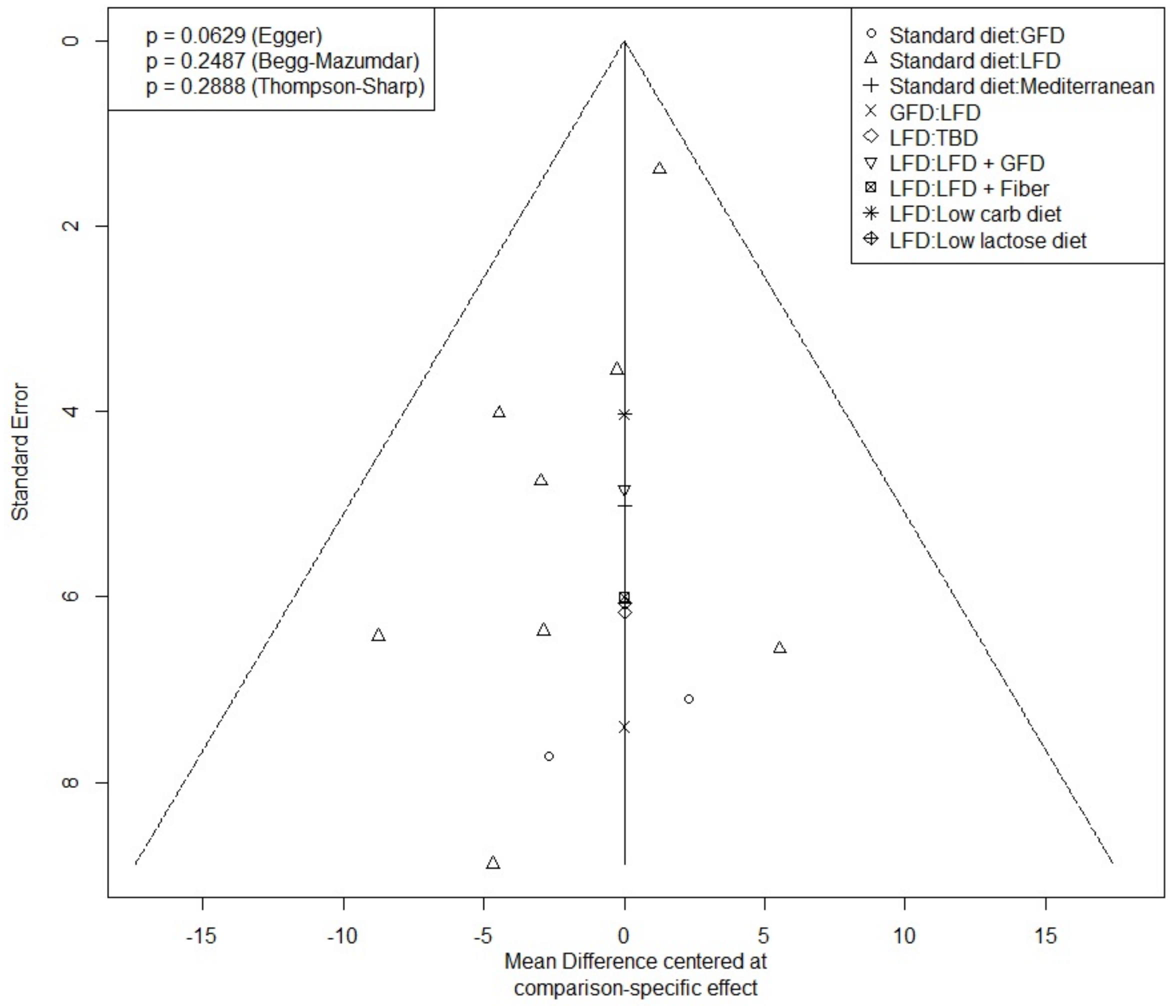


**Supplementary Figure S9.** Funnel plot of IBS-SSS (A) IBS-QOL (B), abdominal distension (C), dissatisfaction with bowel habits (D), stool frequency (E), and general life interference (F). (IBS QOL: irritable bowel syndrome quality of life score, IBS-SSS: irritable bowel syndrome symptoms severity scale).
